# Evolutionary history of Otophysi (Teleostei), a major clade of the modern freshwater fishes: Pangaean origin and Mesozoic radiation

**DOI:** 10.1186/1471-2148-11-177

**Published:** 2011-06-22

**Authors:** Masanori Nakatani, Masaki Miya, Kohji Mabuchi, Kenji Saitoh, Mutsumi Nishida

**Affiliations:** 1 Atmosphere and Ocean Research Institute, The University of Tokyo, 5-1-5 Kashiwanoha, Kashiwa-shi, Chiba 277-8564, Japan; 2 Natural History Museum and Institute, Chiba, 955-2 Aoba-cho, Chuo-ku, Chiba 260-8682, Japan; 3 National Research Institute of Fisheries Science, 2-12-4 Fukuura, Kanazawa, Kanagawa 236-8648, Japan

## Abstract

**Background:**

Freshwater harbors approximately 12,000 fish species accounting for 43% of the diversity of all modern fish. A single ancestral lineage evolved into about two-thirds of this enormous biodiversity (≈ 7900 spp.) and is currently distributed throughout the world's continents except Antarctica. Despite such remarkable species diversity and ubiquity, the evolutionary history of this major freshwater fish clade, Otophysi, remains largely unexplored. To gain insight into the history of otophysan diversification, we constructed a timetree based on whole mitogenome sequences across 110 species representing 55 of the 64 families.

**Results:**

Partitioned maximum likelihood analysis based on unambiguously aligned sequences (9923 bp) confidently recovered the monophyly of Otophysi and the two constituent subgroups (Cypriniformes and Characiphysi). The latter clade comprised three orders (Gymnotiformes, Characiformes, Siluriformes), and Gymnotiformes was sister to the latter two groups. One of the two suborders in Characiformes (Characoidei) was more closely related to Siluriformes than to its own suborder (Citharinoidei), rendering the characiforms paraphyletic. Although this novel relationship did not receive strong statistical support, it was supported by analyzing independent nuclear markers. A relaxed molecular clock Bayesian analysis of the divergence times and reconstruction of ancestral habitats on the timetree suggest a Pangaean origin and Mesozoic radiation of otophysans.

**Conclusions:**

The present timetree demonstrates that survival of the ancestral lineages through the two consecutive mass extinctions on Pangaea, and subsequent radiations during the Jurassic through early Cretaceous shaped the modern familial diversity of otophysans. This evolutionary scenario is consistent with recent arguments based on biogeographic inferences and molecular divergence time estimates. No fossil otophysan, however, has been recorded before the Albian, the early Cretaceous 100-112 Ma, creating an over 100 million year time span without fossil evidence. This formidable ghost range partially reflects a genuine difference between the estimated ages of stem group origin (molecular divergence time) and crown group morphological diversification (fossil divergence time); the ghost range, however, would be filled with discoveries of older fossils that can be used as more reasonable time constraints as well as with developments of more realistic models that capture the rates of molecular sequences accurately.

## Background

Although freshwater lakes and rivers occupy a small portion of the Earth's surface (0.8%) and hold a negligible amount of the total water on Earth (0.01%), these ecosystems support an extraordinarily high proportion of the world's biodiversity, consisting of at least 100,000 species or nearly 6% of all described species [1]. While this enormous biodiversity has been described through continued efforts by taxonomists, its origin and the history of the diversification on a global scale remain largely unexplored across diverse taxa. This is even true for well-studied taxa such as fishes, which account for the largest proportion of vertebrate diversity and is the taxon that controls the trophic structure of freshwater ecosystems [2].

**Figure 1:**
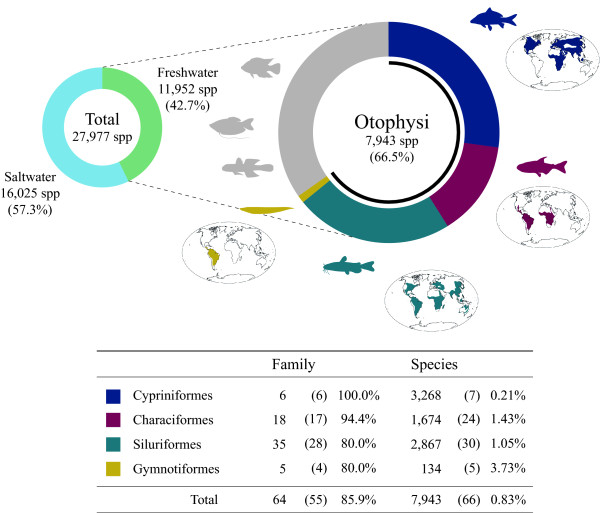
**Species diversity and geographic distributions of otophysans **[3]. Numbers of families and species for the four orders are indicated below the pie charts with those sampled in the present study in parentheses.

Freshwater fishes are disproportionately species-rich. Of the currently recognized 27,977 fish species, 11,952 species (42.7%) occur exclusively in freshwater [3] (Figure 1). These freshwater fishes are polyphyletic, being found in 30 of the 40 orders from the bottom to the top of the ray-finned fish phylogenies [3]. A single ancestral lineage, however, diversified into approximately two-thirds of this enormous diversity (7943 spp.), and it is distributed throughout the world's continents except Antarctica [4]. This clade, Series Otophysi, comprises four primarily freshwater orders (Figure 1): Cypriniformes (minnows, carps, loaches, suckers), Characiformes (tetras, piranhas), Siluriformes (catfishes), and Gymnotiformes (electric eels). These otophysan fishes share modifications of the inner ear, gas bladder, and of the four or five anterior vertebrae and associated elements together called the Weberian apparatus [3]. In a broader phylogenetic context, otophysan fishes represent one of the clades in Subdivision Otocephala [5] along with the orders Gonorynchiformes (= Anotophysi) [6] and Alepocephaliformes [5,7]. Otocephala itself represents a sister clade of Subdivision Euteleostei [8] comprising numerous marine species, including those of Series Percomorpha [3,9,10].

**Figure 2:**
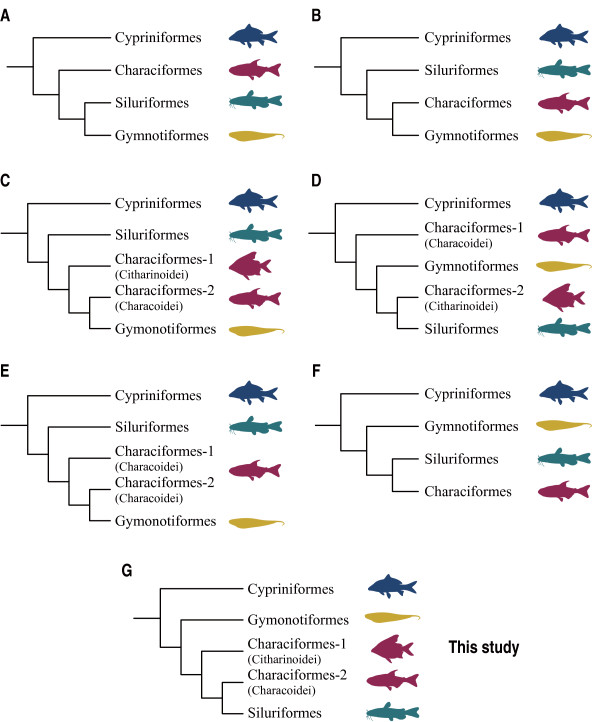
**Alternative phylogenetic hypotheses of the otophysans**. A) Morphology-based hypothesis of Fink and Fink [27] and that of Dimmick and Larson [28] based on combined morphological and molecular data; Molecular-based hypotheses of B) Dimmick and Larson [28] and Saitoh et al. (maximum likelihood analysis) [4]; C) Ortí and Meyer [29]; D) Ortí and Meyer [19]; E) Saitoh et al. (maximum parsimony analysis; the study includes only two characoids) [4] and Peng et al. [30]; F) Lavoué et al. [6] and Poulsen et al. [5] and Li et al. [31]; and G) this study.

Although recent molecular phylogenetic studies have increased our understanding of the interfamilial relationships within each of the four otophysan orders remarkably (Cypriniformes [11-18]; Characiformes [19,20]; Siluriformes [21,22]; Gymnotiformes [23,24]), few studies have specifically addressed the interordinal relationships of the otophysans. Following the advent of cladistic methods [25,26], Fink and Fink [27] proposed the first explicit hypothesis of the otophysan phylogenies (Figure 2A) based on examinations of 127 morphological characters. Subsequently Dimmick and Larson [28] corroborated this hypothesis based on the combined morphological and molecular data (nuclear and mitochondrial rDNA sequences), although the molecular data alone provided a different hypothesis (Figure 2B). The latter hypothesis is congruent with that reported by Saitoh et al. [4] who performed a maximum likelihood (ML) analysis using whole mitogenome sequences from major otophysan lineages. Monophyly of Characiformes, however, was not recovered in the two publications of Ortí and Meyer, who analyzed characiphysan phylogenies using nuclear ependymin [29] (Figure 2C) and mitochondrial rDNA sequences [19] (Figure 2D), which is also true for Peng et al. [30] and the results of maximum parsimony analysis reported by Saitoh et al. [4] but in a different manner (Figure 2E). Notably, more recent molecular phylogenetic studies based on both whole mitogenomes [5,6] and nuclear genes [31] have converged to an additional hypothesis (Figure 2F; but see [24]), although these studies did not specifically address the resolution of otophysan phylogenies. Thus, no consensus exists regarding the relationships among major otophysan lineages with the exception of the most basal position of Cypriniformes. Nevertheless, these molecular studies lacked sufficient taxonomic and/or character sampling (Table 1) to resolve higher-level relationships among otophysan lineages, which exhibit enormous taxonomic diversity (Figure 1). Actually none of the previous studies sampled longer nucleotide sequences (e.g., >5000 bp) from more than three species across all of the four orders (Table 1).

**Table 1:** A summary of character and taxon sampling in the previous molecular phylogenetic studies that include the four otophysan orders

Study	Figure 2	**Marker**^**a**^	Length (bp)	Cyp	Gym	Cha		Sil	Total
								
						Cith	Char		
Dimmick & Larson [28]	B	nc + mt rDNA	2477	2	2	1	1	3	9
Ortí & Meyer [29]	C	nc ependymin	588	3	2	1	12	4	22
Ortí & Meyer [19]	D	mt rDNA	870	2	3	2	11	4	22
Saitoh et al. [4]	B,E	mt genome	8096	7	2	0	2	2	13
Lavoué et al. [6]	F	mt genome	10395	7	2	0	2	2	13
Peng et al. [30]	E	mt genome	6198	8	2	0	2	5	17
Li et al. [31]	F	10 nc genes	7995	3	1	0	1	1	6
Poulsen et al. [5]	F	mt genome	11076	14	2	0	2	5	23
Alves-Gomes [24]	A	mt rDNA	701	15	15	2	13	15	60
This study	G	mt genome	9923	7	5	3	21	30	66
This study	G	10 nc genes	7995	3	2	1	2	2	10

Abbreviations for orders and suborders: Cyp = Cypriniformes; Gym = Gymnotiformes; Cha = Characiformes; Sil = Siluriformes; Cith = Citharinoidei; Char = Characoidei

^a ^nc = nuclear; mt = mitochondrial.

In Otophysi, a great deal of attention has been paid to the biogeographic history that has shaped the current distribution patterns. Indeed, contrasting patterns in the geographic distributions of modern otophysans (Gondwanan vs. Laurasian vs. Pangaean distributions; Figure 1), the general acceptance of plate tectonics and continental drift as explanatory factors for dispersal across land connections and/or as a causal mechanism of vicariant speciation, and the presumed designation of the ostariophysans (Otophysi + Gonorynchiformes) as "primary freshwater fishes" (with dispersal across oceans being unlikely) together led many authors to propose evolutionary scenarios that attempt to identify a center of origin and dispersal routes through land connections based on various assumptions [4,12,27,32-37]. Reconstruction of the history of otophysan diversification (e.g., the history of the modern familial diversification), however, remains unchallenged, apparently because of poor representation in the fossil record before the Cenozoic period [24,38-41], a huge extant taxonomic diversity encompassing over 7943 species placed in 64 families and 1068 genera [3], and the absence of an adequate timescale for the phylogenies across major lineages (but see [30]). Indeed, in a review of the early radiation of teleosts, Arratia [42] stated "... the enormous radiation of some modern groups such as otophysans, atherinomorphs, perciforms, etc. is missing a historical framework."

To provide an overview of the history of modern otophysan diversification within the broad context of the evolutionary history of ray-finned fishes (Actinopterygii), we assembled whole mitochondrial genome (mitogenome) sequences from 66 otophysans (including 51 newly determined sequences), representing 55 of the 64 currently recognized families (86%; Figure 1). The 66 sequences were concatenated with those from 44 outgroup species for a total of 110 species and unambiguously aligned sequences (9923 bp excluding quickly saturated third codon positions) were subjected to phylogenetic analysis and a relaxed-clock Bayesian divergence time estimation. The resultant timetree suggests that the modern otophysan diversity has been shaped through the two consecutive mass extinction events on the Pangaea supercontinent and subsequent radiations during the Jurassic through the early Cretaceous.

## Methods

### Taxonomic sampling

In addition to the 54 fish species used in Azuma et al. [43], 66 otophysans were newly added in this study, making a total of 110 species analyzed (Table 2). The 54 outgroup species encompass the whole actinopterygians (ray-finned fishes) from the bottom to the top of the tree and the 66 otophysans include 55 of the 64 currently recognized families (Figure 1).

**Table 2:** List of the species used in this study

**Order/suborder/superfamily**^**a**^	Family	**Species**^**b**^	**Accession No.**^**c**^
**Outgroup**			
Coelacanthiformes	Latimeriidae	*Latimeria menadoensis*	AP006858
Ceratodontiformes	Ceratodontidae	*Neoceratodus forsteri*	AJ584642
Polypteriformes	Polypteridae	*Polypterus ornatipinnis*	AP004351
		*Polypterus senegalus*	AP004352
		*Erpetoichthys calabaricus*	AP004350
Acipenseriformes	Acipenseridae	*Acipenser transmontanus*	AB042837
		*Scaphirhynchus *cf. *albus*	AP004354
	Polyodontidae	*Polyodon spathula*	AP004353
Lepisosteiformes	Lepisosteidae	*Lepisosteus oculatus*	AB042861
		*Atractosteus spatula*	AP004355
Amiiformes	Amiidae	*Amia calva*	AB042952
Hiodontiformes	Hiodontidae	*Hiodon alosoides*	AP004356
Osteoglossiformes	Osteoglossidae	*Osteoglossum bicirrhosum*	AB043025
		*Pantodon buchholzi*	AB043068
Albuliformes	Notacanthidae	*Notacanthus chemnitzi*	AP002975
Anguillformes	Anguillidae	*Anguilla japonica*	AB038556
	Muraenidae	*Gymnothorax kidako*	AP002976
	Congridae	*Conger myriaster*	AB038381
Clupeiformes	Denticipitidae	*Denticeps clupeoides*	AP007276
	Engraulidae	*Engraulis japonicus*	AB040676
	Clupeidae	*Sardinops melanostictus*	AB032554
Gonorynchiformes	Chanidae	*Chanos chaos*	AB054133
	Gonorynchidae	*Gonorynchus greyi*	AB054134
	Kneriidae	*Kneria *sp.	AF007278
	Phractolaemidae	*Phractolaemus ansorgii*	AB070243
Alepocephaliformes	Platytroctidae	*Platytroctes apus*	AP004107
	Bathylaconidae	*Herwigia kreffti*	AP009582
	Alepocephalidae	*Alepocephalus tenebrosus*	AP004100
Salmoniformes	Salmonidae	*Salmo salar*	U12143
Esociformes	Esocidae	*Esox lucius*	AP004103
Polymixiiformes	Polymixiidae	*Polymixia japonica*	AB034826
Gadiformes	Gadidae	*Gadus morhua*	X99772
Perciformes	Cichlidae	*Oreochromis *sp.	AP009126
		*Neolamprologus brichardi*	AP006014
		*Tropheus duboisi*	AP006015
		*Astronotus ocellatus*	AP009127
		*Paretroplus maculatus*	AP009504
		*Etroplus maculatus*	AP009505
		*Hypselecara temporalis*	AP009506
		*Ptychochromoides katria*	AP009507
		*Paratilapia polleni*	AP009508
		*Tylochromis polylepis*	AP009509
Tetraodontiformes	Tetraodontidae	*Takifugu rubripes*	AJ421455
		*Tetraodon nigroviridis*	AP006046
**Ingroup**			
Cypriniformes			
Cyprinoidea	Cyprinidae	*Cyprinus carpio*	X61010
		*Danio rerio*	AC024175
	Psilorhynchidae	*Psilorhynchus homaloptera*	DQ026436
Cobitoidea	Gyrinocheilidae	*Gyrinocheilus aymonieri*	AB242164
	Catostomidae	*Catostomus commersonii*	AB127394
	Cobitidae	*Cobitis striata*	AB054125
	Balitoridae	*Formosania lacustre*	M91245
Characiformes			
Citharinoidei	Distichodontidae	*Distichodus sexfasciatus*	AB070242**
		*Ichthyborus *sp.	AP011993**
	Citharinidae	Citharinus congicus*	AP011985**
Characoidei	Parodontidae	*Parodon affinis*	AP011998**
	Curimatidae	*Curimatopsis evelynae*	AP011988**
	Anostomidae	*Leporinus affinis*	AP011994**
	Chilodontidae	*Chilodus punctatus*	AP011984**
	Crenuchidae	*Crenuchus spilurus*	AP011986**
	Hemiodontidae	*Hemiodopsis gracilis*	AP011990**
	Alestidae	*Micralestes *sp.	AP011996**
		*Phenacogrammus interruptus*	AB054129
	Gasteropelecidae	*Carnegiella strigata*	AP011983**
	Characidae	*Astyanax mexicanus*	AP011982**
		*Chalceus macrolepidotus*	AB054130
		*Myleus *sp.	AP011997**
		*Paracheirodon innesi*	AP011999**
		*Pygocentrus nattereri*	AP012000**
	Acestrorhynchidae	*Acestrorhynchus *sp.	AP011981**
	Cynodontidae	*Hydrolycus scomberoides*	AP011989**
	Erythrinidae	*Hoplias malabaricus*	AP011992**
	Lebiasinidae	*Lebiasina astrigata*	AP011995**
	Ctenoluciidae	*Boulengerella maculata*	AB070207
		Ctenolucius hujeta*	AP011987**
	Hepsetidae	*Hepsetus odoe*	AP011991**
Siluriformes			
Diplomystoidea	Diplomystidae	*Diplomystes nahuelbutaensis*	AP012011**
Cetopsoidea	Cetopsidae	*Cetopsidium *sp.	AP012007**
		Helogenes marmoratus*	AP012014**
Loricaroidea	Amphiliidae	*Amphilius *sp.	AP012002**
	Tricomycteridae	*Trichomycterus areolatus*	AP012026**
	Callichthyidae	*Corydoras rabauti*	AB054128
	Astroblepidae	*Astroblepus *sp.	AP012004**
	Loricariidae	*Pterygoplichthys disjunctivus*	AP012021**
Sisoroidea	Amblycipitidae	*Liobagrus reinii*	AP012015**
	Erethistidae	*Hara jerdoni*	AP012012**
	Aspredinidae	*Bunocephalus coracoideus*	AP012006**
Cranoglanoidea	Cranoglanididae	*Cranoglanis bouderius*	AY898626
Ictaluroidea	Ictaluridae	*Ictalurus punctatus*	AF482987
Doradoidea	Mochokidae	*Synodontis schoutedeni*	AP012023**
	Doradidae	*Amblydoras gonzalezi*	AP012001**
	Auchenipteridae	*Tatia pergiae*	AP012024**
		*Tetranematichthys quadrifilis*	AP012025**
Siluroidea	Siluridae	*Silurus asotus*	AP012022**
	Malapteruridae	*Malapterurus electricus*	AP012016**
	Auchenoglanididae	*Auchenoglanis occidentalis*	AP012005**
	Chacidae	*Chaca bankanensis*	AP012008**
	Plotosidae	*Plotosus japonicus*	AP012020**
	Clariidae	*Clarias *sp.	AP012010**
	Heteropneustidae	*Heteropneustes fossilis*	AP012013**
Bagroidea	Claroteidae	*Chrysichthys *sp.	AP012009**
	Ariidae	*Sciades seemanni*	AP012003**
	Schilbeidae	*Pareutropius debauwi*	AP012017**
	Pangasiidae	*Pangasius larnaudii*	AP012018**
	Bagridae	*Pseudobagrus tokiensis*	AB054132
	Pimelodidae	*Pimelodus pictus*	AP012019**
Gymnotiformes			
Gymnotoidei	Gymnotidae	*Electrophorus electricus*	AP011978**
		Gymnotus carapo*	AP011979**
Sternopygoidei	Rhamphichthyidae	*Gymnorhamphichthys *sp.	AP011980**
	Sternopygidae	*Eigenmannia *sp.	AB054131
	Apteronotidae	*Apteronotus albifrons*	AB054132

^a ^Classifications follow [3] except for the recognition of Alepocephaliformes [5,7].

^b ^Those species with single asterisks were also used for nuclear DNA sequence analysis.

^c ^Those sequences with double asterisks were newly determined during this study.

### Specimens and DNA extraction

A portion of epaxial musculature or pectoral fins (~0.25 g) from fresh specimens of each species was excised and the tissue was immediately preserved in 99.5% ethanol. Total genomic DNA from the ethanol-preserved tissue was extracted using DNeasy (Qiagen) and Aquapure genomic DNA isolation kit (Bio-Rad Laboratories, Inc.) in accordance with the respective manufacturer's protocols, or the standard phenol-chloroform method as described in Asahida et al. [44].

### PCR and sequencing

Whole mitogenome sequences of the 51 otophysans (double asterisks in Table 2) were determined using a combination of long and short PCR methods developed by Miya and Nishida [45]. Briefly, the mitogenomes of the 51 otophysans in their entirety were amplified using a long PCR technique [46] in two or three reactions. Dilution of the long PCR products with TE buffer (1:10 to 100 depending on the concentration of the long PCR products) served as templates for subsequent short PCRs. Standard sets of fish-versatile primers (and species-specific primers if necessary) were used for short PCRs to amplify contiguous overlapping segments of the entire mitogenome for each otophysan species. The short PCR products were purified using the Exosap-IT enzyme (GE Healthcare Bio-Sciences Corp.) and subsequently sequenced with dye-labeled terminators (BigDye terminator ver. 1.1/3.1; Applied Biosystems) and the primers used in the short PCRs. Sequencing reactions were conducted according to the manufacturer's instructions, followed by electrophoresis on an ABI Prism 377, 3100, or 3130 DNA sequencer (Applied Biosystems). A list of PCR primers used in this study is available from MNa upon request.

In some cases when multiple bands were amplified during short PCRs, we conducted subcloning using MinElute (Qiagen), pGEM-T Easy Vector Systems (Promega), and Z-competent *E. coli *(ZYMO Research), in accordance with the manufactures' protocols. To avoid PCR errors, we sequenced eight clones for each fragment using SP6 and T7 primers.

### Sequence editing and alignment

Mitogenome sequences from the 66 otophysans were concatenated with the pre-aligned sequences used in Azuma et al. [43] in FASTA format and subjected to multiple alignment using MAFFT ver. 6.707 [47]. The aligned sequences were imported into MacClade ver. 4.08 [48] and the resulting gaps in the aligned sequences were manually removed to correctly reproduce the alignment used by Azuma et al. [43]. All the resulting positions with gaps were removed, so the final data set consisted of 6904 positions from the first and second codon positions of the 12 protein-coding genes (excluding the ND6 gene because of its heterogeneous base composition and poor phylogenetic performance [49]), 1622 positions from the two rRNA genes, and 1397 positions from the 22 tRNA genes (total 9923 positions) (designated as 12_n_RT_n_: where 1, 2, R and T represent 1st codon position, 2nd codon position, rRNA gene and tRNA gene, respectively, and the subscript "n" denotes nucleotides). The third codon positions of the protein-coding genes were excluded from the data set because of the extremely high substitution rates (and the resulting multiple hits) and heterogeneous base composition as sources of systematic noise in phylogenetic analysis at this taxonomic level [49,50] and overestimation of divergence time [51,52]. The aligned sequences are available from TreeBase with the following URL (http://purl.org/phylo/treebase/phylows/study/TB2:S11469).

To investigate the relationships within the otophysans, we also created an additional four data sets that treated 12 protein-coding genes differently. The first three data sets considered only transversional changes in the first and/or third codon positions by converting purine (A/G) and pyrimidine (C/T) nucleotides to A and C, respectively (1_r_2_n_R_n_T_n_, 12_n_3_r_RT_n_, 1_r_2_n_3_r_RT_n_: where the subscript "r" denotes a modified RY-coding following Saitoh et al. [11]). The transitional changes in the first codon positions are somewhat saturated among distantly related taxa [49], and the first data set (1_r_2_n_RT_n_) was expected to reduce phylogenetic noise from the original data set (12_n_RT_n_). The second data set (12_n_3_r_RT_n_) added the RY-coded third codon positions to the original data set, which was expected to increase phylogenetic signals and was predominantly used to resolve interrelationships within the Cypriniformes [11,12], one of the major otophysan clades (Figure 1). The third data set (1_r_2_n_3_r_RT_n_) removed transitional changes in the first codon positions from the second data set. The last data set converted protein-coding genes into amino acids (designated as 123_a_RT_n_) to explore the utility of these sequences in resolving otophysan taxa. Only 66 otophysans plus 12 outgroup species (4 gonorynchiforms + 3 clupeiforms + 3 alepocephaliforms + 2 anguillifoms) were used in these additional data sets to minimize the computation time.

### Phylogenetic analysis

Unambiguously aligned sequences were divided into three to five partitions depending on the data sets (three partitions in the 123_a_RT_n _data set, four partitions in the 12_n_RT_n _and 1_r_2_n_RT_n _data sets, and five partitions in the 12_n_3_r_RT_n _and 1_r_2_n_3_r_RT_n _data sets) and subjected to ML analysis. We used RAxML ver. 7.2.8 [53] because it is the only ML-based software that can handle large data sets with data partitioning. A general time reversible model (GTR) [54] with sites following a discrete gamma distribution (Γ) and some sites invariable (I) was selected as the best model of nucleotide sequence evolution by Modeltest ver. 3.7 [55] using the Akaike information criterion (AIC). For amino acid sequences, the MTREV model [56] with sites following a discrete gamma distribution (Γ) and some sites invariable (I) was used. We performed a rapid bootstrap (BS) analysis using this model (GTR + Γ + I) with 1000 replications (-f a option). This performs BS analysis using GTRCAT, which is a GTR approximation with optimization of individual per-site substitution rates and classification of these individual rates into a certain number of rate categories. After implementing the BS analysis, the program uses every fifth BS tree as a starting point for another ML search using the GTR + Γ + I model of sequence evolution and saves the top 10 best-scoring ML trees (fast ML searches). Finally, RAxML calculates more correct likelihood scores (slow ML searches) for those 10 trees and puts BS probabilities (BSPs) on the best-scoring ML tree.

### Evaluation of alternative hypotheses

We manually created the constrained tree topologies with reference to the alternative hypotheses using MacClade and then performed RAxML analysis with each constraint using the -g option. We conducted fast bootstrapping with 100 replicates as described above, and the resulting best-scoring ML tree was considered as the constrained ML tree. The constrained and unconstrained ML trees (best-scoring ML tree without constraint) were used to compute the per-site log likelihood scores for each tree using the -f g option in RAxML and the output was subjected to CONSEL [57] analysis to calculate statistical significance of the differences in likelihood scores. Probabilities of alternative phylogenetic hypotheses were calculated using the likelihood-based approximately unbiased (AU) test [58] as implemented in CONSEL v.0.1k [57]. The *P*-values from this test are calculated using the multi-scale bootstrap technique and are less biased than those of conventional methods [57] such as the BS probability (BSP) [59], the Kishino-Hasegawa (KH) test [60] and the Shimodaira-Hasegawa (SH) test [61].

### Supplementary analysis using nuclear genes

To corroborate the novel relationships among major otophysan lineages obtained in this study (see below), we determined some of the putative single-copy nuclear gene sequences for the selected four otophysans (asterisks in Table 2) according to the methods described by Li et al. [31] (Table 3). We have added these sequences to the aligned data set used in Li et al. (available from http://www.treebase.org/treebase-web/home.html; newID: M3165) who studied actinopterygian phylogenies with 56 species (including three cypriniforms and three characiphysans), and the data set was subjected to partitioned ML analysis according to Kawahara et al. [62].

**Table 3:** DNA accession numbers of the nuclear genes from the four characiphysans

Gene	*Citharinus congicus*	*Ctenolucius hujeta*	*Helogenes marmoratus*	*Gymnotus carapo*
zic1	AB605470	AB605476	--------	AB605488
myh6	--------	--------	--------	--------
PYR3	--------	--------	--------	--------
ptr	AB605467	AB605473	--------	AB605484
Tbr	AB605469	AB605471	AB605480	AB605487
ENC1	AB605465	AB605474	AB605477	AB605481
Glyt	--------	--------	--------	AB605482
SH3PX3	--------	AB605474	--------	AB605485
plagl2	AB605466	AB605472	AB605478	AB605483
sreb2	AB605468	AB605475	AB605479	AB605486

### Divergence time estimation

A relaxed molecular clock Bayesian method implemented in the MCMCTREE program in PAML 4.4b [63] was used for dating analysis. We also attempted to use BEAST [64] for our data set, but MCMC samples failed to converge after 10^8 ^chains. The best-scoring ML tree from the 12_n_RT_n _data set was used for divergence time estimation. The ML estimates of branch lengths were obtained using BASEML and CODEML (in PAML) programs under the GTR + Γ_5 _and MTREVF + Γ_5 _substitution models [54] for the 12_n_RT_n _and 123_a _data sets, respectively, with the gamma prior set at 0.5. Two priors, the overall substitution rate (rgene gamma) and rate-drift parameter (sigma2 gamma), were set at *G *(1, 12.3) and *G *(1, 4.45) for the 12_n_RT_n _data set and *G *(1, 14.3) and *G *(1, 4.5) for the 123_a _data set, respectively, using the strict molecular clock assumption with 445 Ma constraint to the divergence between Actinopterygii and Sarcopterygii (average of the upper and lower constraints for the node between ray-finned and lobe-finned fish; see Table 4). The independent-rates (IR) model [65] was used to specify the prior of rates among internal nodes (clock = 2 in MCMCTREE). The IR model has been considered more appropriate in divergence time estimation than the autocorrelated-rates (AR) model in recent studies (see [66] and references therein), although additional analyses using AR model (clock = 3 in MCMCTREE) were also performed for comparison. The parameters of the birth-death process for tree generation with species sampling [67] were fixed at *λ *= *μ *= 1 and *ρ *= 0, so that the priors are similar to those used in the previous mitogenomic studies [43,68] using MULTIDIVTIME [69]. A loose maximum bound for the root was set at <10.0 (= 1000 Ma).

**Table 4:** Time constraints used for divergence time estimation

Node	Constraints	Calibration information
A	U 472	The minimum age for the basal split of bony fish based on the earliest known acanthodian remains from Late Ordovician [128]
	L 419	The †*Psarolepis *fossil (sarcopterygians) [129] from Ludlow (Silurian) [100]
B	U 419	The minimum age for the Sarcopterygii/Actinopterygii split
	L 392	The †*Moythomasia *fossil (actinopteran) from the Givetian/Eifelian boundary [100]
C	U 392	The minimum age for the Polypteriformes/Actinopteri split
	L 345	The †*Cosmoptychius *fossil (neopterygian or actinopteran) from Tournasian [100]
D	L 130	The †*Protopsephurus *fossil (Polyodontidae) from Hauterivian (Cretaceous) [100]
E	L 284	The †*Brachydegma *fossil (stem amiids) from Artinskian (Permian) [100]
F	L 136	The †*Yanbiania *fossil (Hiodontidae) from the Lower Cretaceous [100]
G	L 112	The †*Laeliichthys *fossil (Osteoglossidae) from the Aptian (Cretaceous) [130]
H	L 151	The †*Anaethalion*, †*Elopsomolos*, and †*Eoprotelops *fossil (Elopomorpha) from Kimmeridgian (Jurassic) [100]
I	L 94	The †*Lebonichthys *(Albulidae) fossil from the Cenomanian (Cretaceous) [130]
J	L 49	The *Conger *(Congridae) and *Anguilla *(Anguillidae) fossils from the Ypresian (Tertiary) [130]
K	L 146	The †*Tischlingerichthys *fossil (Ostariophysi) from Tithonian (Jurassic) [100]
L	L 56	The †*Knightia *fossil (Clupeidae) from the Thanetian (Paleogene) [130]
M	L 49	The †*Parabarbus *fossil (Cyprinidae) from the Ypresian (Paleogene) [130]
N	L 8	Gymnotiform fossil from the Late Miocene [131]
O	L 98	The *Santanichthys *(Characiformes) fossil from Albian (Cretaceous) [94]
P	L 74	The ariid fossil from Campanian (Cretaceous) [132]
Q	L 74	The †*Esteseox foxi *fossil (Esociformes) from the Campanian (Cretaceous) [133]
R	L 94	The †*Berycopsis *fossil (Polymixiidae) from the Cenomanian (Cretaceous) [130]
S	L 98	The tetraodontiform fossil from the Cenomanian [134]
T	L 32	The estimated divergence time between *Takifugu *and *Tetraodon *[135]
U	U 95 L 85	The upper and lower bounds of separation between Madagascar and Indian [104]
V	U 145 L 112	The upper and lower bounds of separation between Indo-Madagascar landmass and Gondwanaland [104]
W	U 120 L 100	The upper and lower bounds of separation between African and South American landmasses [104]

Maximum (U) and minimum (L) time constraints in million years ago (Ma) at nodes depicted in Additional file 1

The MCMCTREE program allows for minimum (lower) and maximum (upper) time constraints, and multiple calibration points have been argued to provide overall more realistic divergence time estimates [70]. Therefore we sought to obtain optimal phylogenetic coverage of calibration points across our tree, although we could set maximum constraints based on the fossil records only for the six nodes (Table 4). Other than these six nodes, 17 additional nodes were reasonably chosen to constraint their minimum ages only (total 29 time constraints for 23 nodes; Table 4). A hard and softbound version of the program (MCMCTREE-HS) was used, so that probabilities of the true divergence time falling outside the minimum bounds are zero, but small but not zero for the maximum bounds [71]. All time constraints are provided in units of 100 Ma (i.e., 1 = 100 Ma) because some of the model components in the Bayesian analysis are scale-variant [63]. The calibration nodes with minimal bound only were set as *L *(*t*_min_) and those with both minimal and maximal bounds were set as *B *(*t*_min_, *t*_max_). The former setting (*L*) assumes a heavy-tailed density (nearly a flat prior) based on a truncated Cauchy distribution of *p *= 0.1 and *c *= 1 as the default [63] ("standard minimum-age constraints" [72]). We did not manipulate the two shape parameters of the truncated Cauchy distribution because of insufficient information with which to specify meaningful prior distributions for most otophysan diversification times.

MCMC approximation with a burn-in period of 10,000 cycles was obtained, and every 50 cycles was taken to create a total of 10,000 samples. To diagnose possible failure of the Markov chains to converge to their stationary distribution, we performed two replicate MCMC runs with two different random seeds for each analysis. MCMC samples from the two runs were combined after checking the distributions of parameter values using Tracer 1.5 (available from http://tree.bio.ed.ac.uk/software/tracer/). The number of samples (20,000) was large enough to reach effective sample sizes (ESSs >200) for all parameters estimated in this study.

To evaluate the effects of topological uncertainties on divergence time estimation, we also conducted dating analyses with four of the 14 alternative topologies (four topologies with the second best *P *value, the two worst *P *values, plus a hypothesis advocated by Fink and Fink [27]).

### Tracing character evolution

The ancestral habitat was reconstructed on the timetree under a ML optimality criterion using Mesquite ver. 2.71 [73]. The ML reconstruction methods found that the ancestral states maximizing the probability of the observed states would evolve under a stochastic model of evolution [74,75]. The Mk1 model ("Markov k-state 1 parameter model"), a k-state generalization of the Jukes-Cantor model that corresponds to Lewis' Mk model [76], was used to trace the character evolution. Two character states were assigned to the terminal node: saltwater (character state 0) and freshwater (state 1).

## Results and Discussion

### Genome organization

Complete L-strand nucleotide sequences from the mitogenomes of the 51 species newly determined during this study were deposited in the DNA Data Bank of Japan (DDBJ), European Molecular Biology Laboratory (EMBL), and GenBank (Table 2). The genome content of the 51 species included two rRNA, 22 tRNA, and 13 protein-coding genes, plus the putative control region, as found in other vertebrates. Their gene arrangements were identical to the typical gene order of vertebrates.

### Interordinal/subordinal relationships

**Figure 3:**
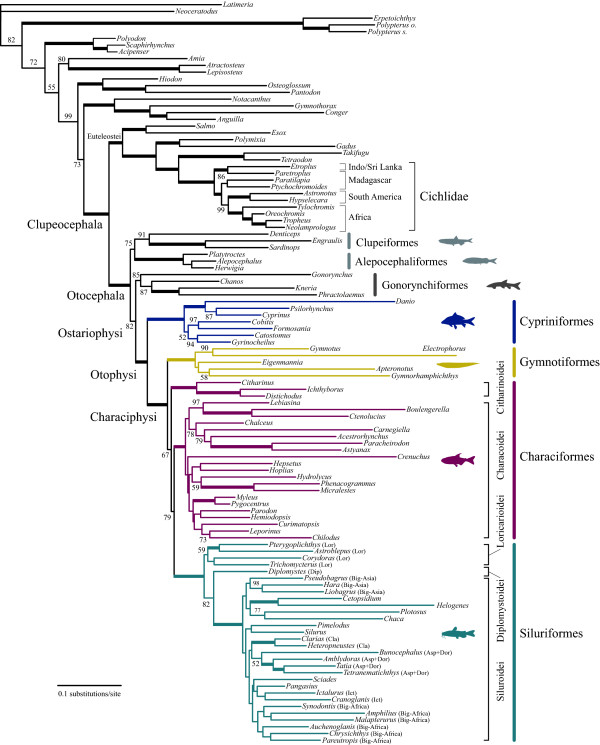
**The best-scoring maximum-likelihood (ML) tree of the 66 otophysan and 44 outgroup species based on unambiguously aligned whole mitogenome sequences (12_n_RT_n _data set; 9923 positions)**. Numerals beside internal branches indicate bootstrap probabilities (BSPs) of ≥50% based on 1000 replicates. Thick lines indicate those internal branches with 100% BSPs. Suprafamilial clades in Siluriformes reported by Sullivan et al. [21] are denoted as follows (abbreviations in parentheses): "Big Asia", "Claroidea" (Cla), "Aspredinidae + Doradoidea" (Asp + Dor), "Ictaluroidea" (Ict), and "Big Africa".

Partitioned ML analysis based on 110 whole mitogenome sequences (12_n_RT_n _data set) resulted in a relatively well resolved tree, with approximately 70% of the internal branches supported by moderate to high (70-100%) BSPs (Figure 3). Otocephala was confidently recovered as a monophyletic group (BSP = 100%) and a clade containing two primarily marine orders (Clupeiformes and Alepocephaliformes) was sister to all other otocephalans. Gonorynchiformes was recovered as the sister group of Otophysi (BSP = 82%) and monophyly of the latter was confidently recovered (BSP = 100%; Figure 3), confirming the results of recent molecular studies [5,7,31]. Of the four orders within the otophysans, monophyletic Cypriniformes (BSP = 100%) was a sister to a clade containing the remaining three orders collectively called Characiphysi (BSP = 100%). Of the three characiphysan orders, Gymnotiformes and Siluriformes were confidently recovered as monophyletic groups (BSPs = 100%); however, one of the two suborders of Characiformes (Characoidei) was more closely related to Siluriformes (BSP = 79%) than to the other (Citharinoidei). Statistical support for a clade containing these three lineages (Citharinoidei, Characoidei, Siluriformes), however, was not strong (BSP = 67%).

**Figure 4:**
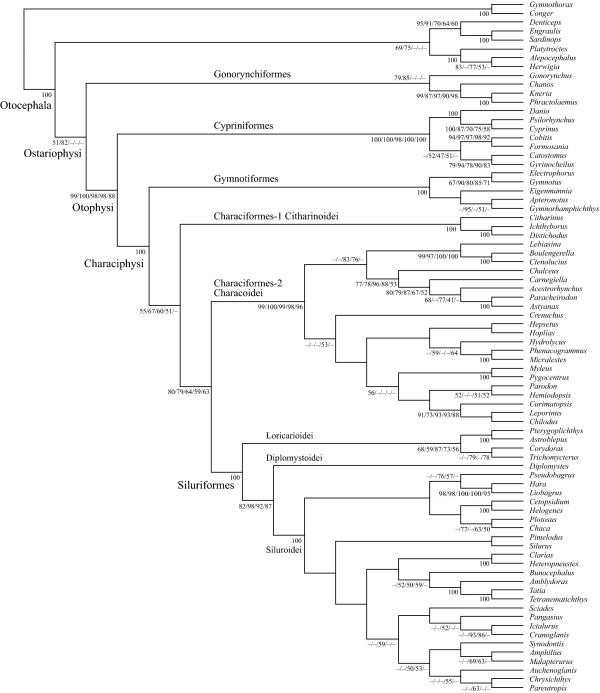
**Bootstrap probabilities (BSPs) from RAxML analyses of the five different data sets that treated codon positions from the 12 protein-coding genes differently**. The tree topology is derived from the RAxML analysis based on limited taxonomic sampling from the 12_n_RT_n _data set (all ingroup species + 12 outgroup species). Numerals beside internal branches indicate BSPs of ≥50% based on 1000 replicates (BSPs of 1_r_2_n_RT_n_/12_n_RT_n_/1_r_2_n_3_r_RT_n_/12_n_3_r_RT_n_/123_a_RT_n _data sets from left to right). Single values denote the same BSPs obtained across the five data sets.

Removal of transitional changes from the 1st codon positions (1_r_2_n_RT_n _data set), addition of transversional changes from the 3rd codon positions (12_n_3_r_RT_n _and 1_r_2_n_3_r_RT_n _data sets), or conversion of the protein-coding genes into amino acid sequences (123_a_RT_n_) together supported the novel relationships, although they did not improve the statistical support (<50-67% BSPs for Citharinoidei + Characoidei + Siluriformes and 59-80% BSPs for Characoidei + Siluriformes) (Figure 4).

**Figure 5:**
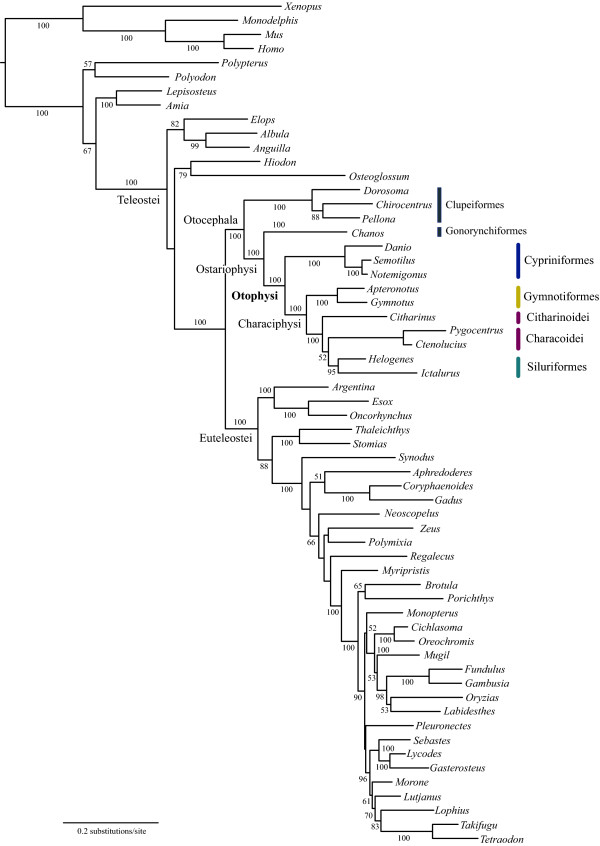
**The best-scoring maximum-likelihood (ML) tree of the 10 otophysan and 50 outgroup species based on unambiguously aligned 10 nuclear gene sequences (8409 positions)**. Numerals beside internal branches indicate bootstrap probabilities (BSPs) of ≥50% based on 1000 replicates.

Although taxonomic sampling from otophysans in the nuclear data set was far sparser than that of the mitogenomic data set (10 spp. vs. 66 spp.), and data were missing in the original as well as newly added sequences (approximately 25% of the total positions; Table 3), partitioned ML analysis based on the 10 nuclear genes from the 60 species resulted in an identical tree topology to that of the mitogenomic tree regarding the relationships among the major otophysan lineages (Figure 5). Monophyly of Otophysi was confidently recovered at the apical position in Otocephala (BSP = 100%), with Cypriniformes being a sister to all other otophysans. Within the Characiphysi, Gymnotiformes was a sister to other characiphysans and monophyly of Characiformes plus Siluriformes was supported with 100% BSP (67% BSP in mitogenomic analysis; Figure 3), although the former was found to be paraphyletic, with Characoidei being more closely related to Siluriformes (BSP = 52%) than to Citharinoidei.

Paraphyletic characiforms have been repeatedly recovered in previous molecular studies (Figure 2C-E) but in different manners. For example, Ortí and Meyer [29] assembled nuclear ependymin sequences (588 bp) from 22 otophysans to explore the phylogenetic utility of this gene and found that characoids were more closely related to Gymnotiformes than to a citharinoid (*Distichodus*) (Figure 2C). To investigate the characiform radiation, Ortí and Meyer [19] used partial mitochondrial rDNA sequences (870 bp) as phylogenetic markers and observed that two citharinoids (*Distichodus *and *Citharinus*) were more closely related to Siluriformes than to characoids (Figure 2D). Although analyses of whole mitogenome sequences by Saitoh et al. [4] (maximum parsimony analysis of 8096 bp) and Peng et al. [30] (6198 bp) did not include citharinoids, one of the two characoids were more closely related to either Siluriformes or Gymnotiformes than to its own order (Figure 2E). These relationships, however, received weak statistical support (<50% BSPs) and alternative tree topologies could not be rejected [4,19,29].

Monophyly of Characiformes should be addressed in relation to the other two characiphysan orders. Accordingly, we evaluated the likelihoods of alternative hypotheses among four major characiphysan lineages (Gymnotiformes, Citharinoidei, Characoidei, Siluriformes) using the AU test (Table 5). Of the 15 possible tree topologies based on the mitogenomic data, AU tests confidently rejected only three that commonly included the monophyly of Characoidei plus Gymnotiformes (*P *= 0.001-0.030; Table 5), with likelihoods of the other 12 topologies ranging from 0.051 to 0.702. Moreover, AU tests based on the nuclear data more confidently rejected 12 tree topologies (*P *= 0.000-0.006; Table 6). For either marker, however, AU tests could not reject all of those topologies that included monophyletic Characiformes, and this issue therefore remains unanswered even in the molecular phylogenetic context. Apparently the two deep-branching lineages in the characiforms (Citharinoidei and Characoidei) make it difficult to resolve their relationships with Siluriformes even with the large data sets used in this study. Also we expect that future uses of newly developed models accounting for site-specific modulations of the amino-acid replacement process, such as the CAT mixture model [77] implemented in PhyloBayes [78], may result in more clear resolution of the characiphysan phylogenies. Note that monophyletic Characiformes was well accepted and seemingly uncontroversial on a morphological basis [27,79-81].

**Table 5:** Results from AU-tests among 15 alternative tree topologies of the four major lineages derived from analysis of whole mitogenome sequences

**Tree**^**a**^	ln *L*	Diff -ln *L*	***P ***^**b**^
1(Gym, (Cit, (Cha, Sil)))	-237742.581	best	0.702
2(Gym, (Sil, (Cit, Cha)))	-237746.644	4.063	0.588
3((Cit, Gym), (Cha, Sil))	-237749.618	7.037	0.473
4(Gym, (Cha, (Cit, Sil)))	-237749.793	7.212	0.400
5(Cha, (Sil, (Cit, Gym)))	-237752.770	10.189	0.400
6(Cit, (Gym, (Cha, Sil)))	-237752.584	10.003	0.362
7(Cit, (Cha, (Sil, Gym)))	-237755.898	13.317	0.267
8(Cha, (Cit, (Sil, Gym)))	-237755.375	12.794	0.140
9((Cit, Cha), (Sil, Gym)))	-237758.976	16.395	0.140
10(Cha, (Gym, (Cit, Sil)))	-237756.070	13.489	0.129
11(Sil, (Cha, (Cit, Gym)))	-237760.049	17.468	0.069
12(Sil, (Gym, (Cit, Cha)))	-237764.849	22.268	0.051
13(Cit, (Sil, (Cha, Gym)))	-237761.012	18.431	0.030*
14((Cha, Gym), (Cit, Sil))	-237765.117	22.536	0.009*
15(Sil, (Cit, (Cha, Gym)))	-237765.958	23.377	0.001*

^a ^Gym: Gymnotiformes; Cit: Citharinoidei; Cha: Characoidei; Sil: Siluriformes.

^b ^Statistically significant differences (≤ 0.05) denoted by asterisks.

**Table 6:** Results from AU-tests among 15 alternative tree topologies of the four major lineages derived from analysis of 10 nuclear gene sequences

**Tree**^**a**^	ln *L*	Diff -ln *L*	***P ***^**b**^
1(Gym, (Cit, (Cha, Sil)))	-131608.5	best	0.723
2(Gym, (Cha, (Cit, Sil)))	-131610.3	1.758	0.447
3(Gym, (Sil, (Cit, Cha)))	-131612.0	3.495	0.200
4((Cit, Cha), (Sil, Gym)))	-131649.9	41.379	0.006*
5(Cha, (Gym, (Cit, Sil)))	-131633.8	25.322	0.005*
6(Cit, (Gym, (Cha, Sil)))	-131644.2	35.689	0.004*
7((Cha, Gym), (Cit, Sil))	-131633.6	25.100	0.003*
8(Cha, (Sil, (Cit, Gym)))	-131657.8	49.233	0.002*
9(Sil, (Gym, (Cit, Cha)))	-131656.9	43.761	0.001*
10(Cha, (Cit, (Sil, Gym)))	-131656.9	45.503	0.001*
11(Cit, (Cha, (Sil, Gym)))	-131654.0	45.504	0.001*
12(Sil, (Cit, (Cha, Gym)))	-131656.9	48.413	0.001*
13(Cit, (Sil, (Cha, Gym)))	-131656.9	48.413	0.001*
14((Cit, Gym), (Cha, Sil))	-131644.2	35.662	0.000*
15(Sil, (Cha, (Cit, Gym)))	-131657.9	49.336	0.000*

^a ^Gym: Gymnotiformes; Cit: Citharinoidei; Cha: Characoidei; Sil: Siluriformes.

^b ^Statistically significant differences (≤ 0.05) denoted by asterisks.

### Interfamilial relationships

Interfamilial relationships within each of the four major lineages are outside the scope of this study because taxonomic sampling is still sparse and requires at least several distantly related genera from the same family to reconstruct more reliable trees. Nevertheless, brief comparisons with previous studies are useful to direct future research.

In Cypriniformes, monophyly of one of the two superfamilies (Cyprinoidea) was consistently recovered, while another superfamily (Cobitoidea) has been variously recovered as mono- or paraphyletic depending on the data treatment (most notably that of the third codon position) even within some of the same studies [11-17]. An enigmatic *Psilorhynchus *(placed in its own family Psilorhynchidae) is nested within the cyprinid lineage (*Cyprinus *+ *Danio*; Figure 3), rendering Cyprinidae paraphyletic as reported previously [15,17,18].

In Gymnotiformes, a sister group relationship between two gymnotids (*Electrophorus *and *Gymnotus*) was confidently recovered (BSP = 90%), but other portions of the tree were weakly supported (BSPs ≤58%; Figure 3) and do not warrant further comment. Such ambiguous interfamilial relationships have been reported in previous studies based on mitochondrial rDNA sequences [23,24].

In Citharinoidei, one of the two suborders of Characiformes, two distichodontid genera (*Distichodus *and *Ichthyborus*) formed a strongly supported monophyletic group (BSP = 100%), which was sister to a citharinid (*Citharinus*). These relationships have been consistently recovered in previous studies based on both morphological [79,82] and molecular [19,20,83] analyses. Within Charachoidei, another suborder of Characiformes, basal relationships were poorly resolved and only four of the 19 internal branches that connect two species from the same families received the highest statistical support (100% BSPs; Figure 3). With the exception of such ambiguous basal relationships, the resulting phylogenies showed several similarities to those reported by Calcagnotto et al. [20], who analyzed four nuclear and two mitochondrial genes across 124 characiform taxa. In particular, we confirmed that the two African lineages represented by Hepsetidae (*Hepsetus*) and Alestidae (*Phenacogrammus *and *Micralestes*) did not form a monophyletic group, but were nested in the same clade with strictly Neotropical species, such as *Hydrolycus *(Cynodontidae) and *Hoplias *(Erythrinidae) (Figure 3).

In Siluriformes, monophyly of the two currently recognized suborders (Loricaroidei, Siluroidei [21]) was confirmed and the Loricaroidei was recovered as sister to other clades, followed by the divergence of Diplomystoidei and Siluroidei (BSP = 82%; Figure 3). These basal divergences are fully congruent with the results of a recent molecular phylogenetic study by Sullivan et al. [21], who analyzed two nuclear genes across 110 catfish species representing 36 of 37 families. Thus, the two different lines of evidence support the most basal Loricaroidei in the siluriform phylogenies. Note that previous morphological studies have consistently argued the most basal Diplomystoidei ([41,84-89]), which was supported by a molecular study by Hardman [22], who used *Diplomystes mesembrinus *(Diplomystidae) as the only outgroup to root the tree. Within the Loricaroidei, a sister group relationship between *Pterygoplichthys *(Loricaridae) and *Astroblepus *(Astroblepidae) was strongly supported as in previous morphological and molecular studies [21,89]. As in the Characoidei, basal relationships in the Siluroidei were poorly resolved and only five of the 23 internal branches received high statistical support (≥95% BSPs; Figure 3). Nevertheless we recovered some of the suprafamilial clades reported by Sullivan et al. [21], and list them below with genera used in this study (Figure 3): "Big Asia" (*Pseudobagrus*, *Hara*, *Liobagrus*), "Claroidea (Cla)" (*Clarias*, *Heteropneustes*), "Aspredinidae + Doradoidea (Asp + Dor)" (*Bunocephalus*, *Amblydoras*, *Tatia*, *Tetranematichthys*), "Ictaluroidea (Ict)" (*Ictalurus*, *Cranoglanis*), and "Big Africa" (*Synodontis*, *Amphilius*, *Malapterurus*, *Auchenoglanis*, *Chrysichthys*, *Pareutropius*).

### Estimation of divergence time

MCMCTREE analyses of the divergence times based on the two data sets (12_n_RT_n _and 123_**a**_) with the assumption of independent rates (IR) across nodes yielded similar estimated node ages (see Additional file 1). Overall dating analysis based on the nucleotide data set (12_n_RT_n_) provided slightly older node ages (absolute differences in posterior means: 16.3 million years ± 21.5 SD) with consistently smaller 95% credible intervals (absolute differences: 30.3 million years ± 23.0 SD) than those from the amino acid data set (123_**a**_). Consequently the 95% credible intervals from both data sets greatly overlapped with each other across all nodes. These tendencies held true for the estimated node ages from the most recent common ancestors (MRCAs) of the five major otophysan lineages themselves (Cypriniformes, Gymnotiformes, Citharinoidei, Characoidei, Siluriformes) and those from MRCAs comprising subsets of these five lineages (total = nine nodes), with the absolute difference in posterior means of 17.8 million years ± 5.7 SD. Considering the long evolutionary history of actinopterygians (≈ 450 million years), the differences seem minor and the following description and discussion of the results were based on the nucleotide data set (12_n_RT_n_) for simplicity.

**Figure 6:**
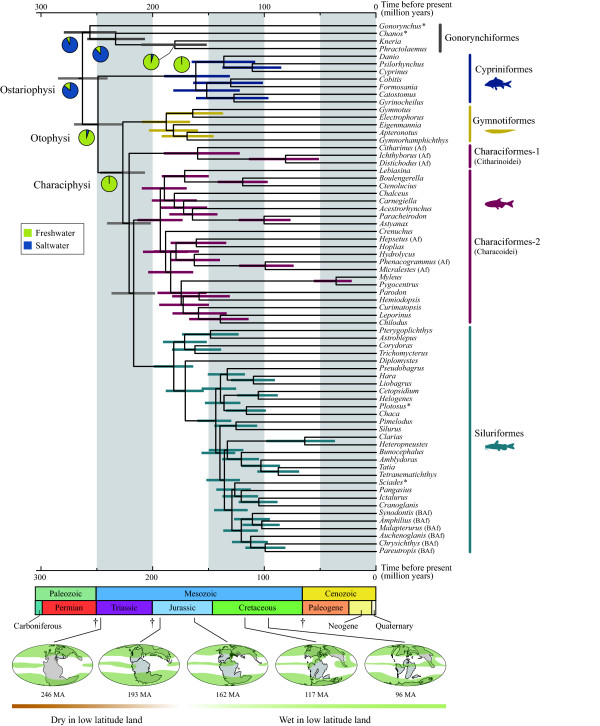
**Timetree derived from the Bayesian relaxed-molecular clock method (only ostariophysan portions shown)**. Horizontal bars indicate 95% credible intervals of the divergence time estimates. ML reconstruction of ancestral habitats are indicated on selected nodes with pie charts showing the likelihoods for two character states (blue, freshwater; light green, saltwater). African characiforms and Big African clade in Siluriformes reported by Sullivan et al. [21] are denoted by "Af" and "BAf", respectively. All marine species are indicated by asterisks. Dagger symbols indicate the three big mass extinction events after 300 Ma. Paleocoastline maps [126] are shown below the timetree with moist zones schematically illustrated by green [127].

MCMC analysis based on the 12_n_RT_n _data set indicated that an ancestral lineage of the modern otophysans diverged from a stem ostariophysan during the end-Permian about 261 Ma with a 95% credible interval of 240-282 Ma (Figure 6). Basal otophysan divergences that produced common ancestors of the five modern lineages were estimated to occur in a relatively short time window during the Triassic (251-200 Ma): divergences between Cypriniformes and Characiphysi = 248 Ma (with a 95% credible interval of 227-268 Ma); Gymnotiformes and Characiformes + Siluriformes = 226 Ma (206-245 Ma); Citharinoidei and Characoidei + Siluriformes 220 Ma (201-240 Ma), Characoidei and Siluriformes 216 Ma (198-237 Ma). The origins of these ancestral lineages were followed by the onset of radiations in each lineage during the Jurassic (200-146 Ma), with the estimated ages of the most recent common ancestor (MRCA) of the modern Cypriniformes = 160 Ma (130-186 Ma); Gymnotiformes = 189 Ma (166-212 Ma); Citharinoidei = 160 Ma (124-190 Ma); Characoidei = 192 (172-213 Ma); and Siluriformes = 180 Ma (162-198 Ma). For the summary of MCMC samples for node ages and entire timetree including non-otophysans, see Additional files 2 and 3, respectively.

We confirmed that uncertainties in relationships among major characiphysan lineages (Gymnotiformes, Citharinoidei, Characoidei, Siluriformes) least affect divergence time estimates, with the largest difference being only 10 million years (Table 7). In addition, despite the use of the IR model across nodes using MCMCTREE in the present study, the resulting ages for selected nodes were very similar to those reported in previous studies employing the AR model using MULTIDIVTIME [43,68] (Table 8). When the AR model was used in MCMCTREE (clock = 3), differences in the resulting node ages were minor, with absolute differences in posterior means of 0.7 million years ± 16.4 SD (Additional file 1). Thus, the topological uncertainties and use of the different models of rate variations across nodes (and associated software) did not affect the divergence time estimates, at least in the present data set.

**Table 7:** Comparisons of the divergence time estimates (Ma) for selected nodes from alternative tree topologies

**Node (MRCA)**^**a**^	Topology 1 (*P *= 0.702)	Topology 2 (*P *= 0.588)	Topology 14 (*P *= 0.009)	Topology 15 (*P *= 0.001)	Topology 9 (*P *= 0.140)
Ostariophysi	261 (240-282)	261 (237-282)	260 (236-282)	261 (237-283)	259 (236-281)
Otophysi	248 (227-268)	246 (225-267)	245 (224-266)	245 (223-267)	245 (224-267)
Cypriniformes	159 (130-186)	160 (131-188)	160 (132-187)	159 (131-186)	160 (132-187)
Characiphysi	226 (206-245)	224 (203-244)	220 (200-239)	220 (201-242)	220 (200-242)
Gymnotiformes	189 (166-212)	184 (163-206)	179 (159-198)	180 (157-200)	180 (158-201)
Citharinoidei	160 (124-190)	152 (118-185)	155 (120-186)	154 (120-186)	153 (119-188)
Characoidei	192 (172-213)	190 (171-210)	187 (168-207)	187 (167-210)	188 (169-209)
Siluriformes	180 (162-198)	180 (162-197)	179 (161-197)	180 (162-197)	180 (162-197)

Four topologies with the two best (topologies 1 and 2) and worst (topologies 14 and 15) probabilities of AU tests in Table 3 plus that of Fink & Fink (topology 9) [27]. Upper and lower values are means and ranges of 95% posterior probabilities, respectively.

^a ^Indicated by taxonomic names of the most recent common ancestor (MRCA)

**Table 8:** Comparisons of divergence time estimates (Ma) for basal actinopterygian nodes between the present and previous mitogenomic studies

**Node (MRCA)**^**a**^	This study (Topology 1)	**Setiamarga *et al. ***[68]	**Azuma *et al. ***[43]
Osteichthyes	445 (419-472)	428 (419-442)	429 (417-449)
Actinopterygii	425 (401-449)	411 (398-419)	415 (400-434)
Actinopteri	380 (360-395)	383 (368-392)	384 (370-392)
Neopterygii	362 (339-381)	364 (346-378)	365 (348-378)
Teleostei	334 (312-357)	332 (312-350)	331 (312-348)
Clupeocephala	293 (271-315)	289 (269-310)	288 (268-307)

^a ^Indicated by taxonomic names of the most recent common ancestor (MRCA)

Our age estimates, however, are remarkably older than those indicated in previous reports based on the fossil record [43,68,90-92]. For example, the oldest otocephalan fossil discovered to date was from the Late Jurassic 150 Ma [93], while our estimate was 265 Ma. The oldest otophysan fossil record dates back to the Albian (100-112 Ma) [94], while our estimate was 248 Ma. For all other major otophysan lineages, our molecular estimates (from 160 Ma in Cypriniformes to 220 Ma in Characiformes) are over 100 million years older than those of the fossil records used as minimum constraints (from 8 Ma in Gymnotiformes to 98 Ma in Characiformes; Table 4). Azuma et al. [43] also noted such large differences and plotted minimum time constraints based on the fossil record against molecular time estimates of the corresponding nodes. They found that four data points in the Paleozoic showed a fairly good 1:1 relationship, whereas other points in the Mesozoic were considerably below the line of a 1:1 relationship. They considered that these significant departures from the expected relationships for the Mesozoic fossils may reflect the fact that they do not really represent the oldest fossil for the corresponding lineages.

For such remarkably older molecular estimates, Benton and Ayala [51] noted four pervasive biases that may cause molecular dates to be too old: 1) the calibration dates that are too old based on previous molecular studies; 2) undetected rapidly evolving genes; 3) an ancestral polymorphism that was maintained through a long evolutionary period; and 4) asymmetric distributions of estimated times, with a constrained younger end but an unconstrained older end. As discussed by Azuma et al. [43], whose data set formed the basis of this study, the first factor is not the case in the present study because we did not use calibration dates based on previous molecular studies. The third factor would be applicable when the genomic regions used are under long-term balancing selection, but no mitochondrial genes have been reported to be under such selection. Neither the second factor nor the fourth factor are true in this study because quickly saturated third codon positions and the control region were excluded from the present analysis, and because each mitochondrial gene used here was tested and shown to perform well for dating vertebrate divergences [95]. We agree with the arguments of Azuma et al. [43] as well as those of Benton and Ayala [51], who stated that "careful choice of genes may be a more appropriate strategy (than the larger data strategy), with a focus on long and fast-evolving (yet alignable) sequences" for reliable dating. Whole mitogenome sequences seem to accommodate such requirements.

Recently Peng et al. [30] and Saitoh et al. [12] estimated divergence times of Ostariophysi and Cypriniformes, respectively, based on whole mitogenome sequences. As expected from similarities in the data sets and priors used in the relaxed-molecular clock Bayesian analysis, their estimates generally agreed with those of our study (Table 9). More recently Santini et al. [96] analyzed divergence times for major fish lineages including those of Otophysi to investigate the effects of fish-specific whole-genome duplication events on the radiations of teleost fishes. Their analyses were based on the nuclear RAG1 gene sequences across 227 vertebrates downloaded from the database, and the phylogenetic relationships were reconstructed by constraining the monophyly of several groups to reflect generally accepted phylogenies. They used BEAST [64] to estimate divergence times under a model of uncorrelated but lognormally distributed rates, with soft upper (= maximum) bounds assigned to the prior distributions of 45 fossil calibrations using lognormal distributions. Note that their estimated ages are considerably younger than those in our study and those reported by Peng et al. [30] and Saitoh et al. [12] (Table 9) and thus require explanation.

**Table 9:** Comparisons of divergence time estimates (Ma) for basal actinopterygian nodes between the present and previous mitogenomic studies

**Node (MRCA)**^**a**^	This study (Topology 1)	**Peng *et al. ***[30]	**Saitoh *et al. ***[12]	**Santini *et al. ***[96]
Otocephala	265 (243-286)	279 (264-293)	251 (230-273)	151 (149-153)
Ostariophysi	261 (240-282)	------	239 (218-260)	128 (125-134)
Anotophysi	254 (232-276)	242 (----)	228 (206-250)	------
Otophysi	248 (227-268)	251 (----)	220 (198-241)	------
Cypriniformes	160 (130-186)	183 (----)	156 (136-176)	92 (56-123)
Characiphysi	226 (206-245)	210 (----)	185 (164-207)^b^	------
Gymnotiformes	189 (166-212)	150 (----)	------	------
Characiformes	220 (201-240)	203 (----)	160 (139-183)^b^	80 (68-84)
Siluriformes	180 (162-198)	173 (----)	125 (104-148)^b^	88 (77-98)

^a ^Indicated by taxonomic names of the most recent common ancestor (MRCA)

^b ^Including only two characoid characiforms plus two siluriforms, which are likely to provide underestimation of the node ages owing to insufficient taxonomic sampling.

As expected from the remarkably narrow 95% credible intervals in the estimated ages for Otocephala (149-153 Ma; Table 9), Santini et al. [96] set both minimum (149 Ma) and maximum (152 Ma) time constraints for the MRCA of Ostarioclupeomorpha (= Otocephala in this study). The upper bound (152 Ma) was chosen based on their strong belief that the MRCA of Otocephala was unlikely to be older than the oldest stem elopomorph (true eels and their relatives: †*Anaethalion*) from the late Kimmeridgian, Jurassic 152 Ma [42]. Accordingly, they manipulated three priors of the lognormal distribution (offset = 149; mean = 0.1; SD = 0.6), which allowed only 5% of the MCMC samples to exceed the soft upper bound (152 Ma). This approach is called "phylogenetic bracketing" [97], which obtains not only minimum, but also maximum constraints on the timing of a branching event using the dates of the preceding and subsequent branching episodes [98,99]. Donoghue and Benton [97] argued that phylogenetic bracketing may be problematic because it assumes that the branching events above and below a calibration more reliably capture the timing of the branching event in question than the estimated date of the calibration itself. For other clades within the Otocephala, Santini et al. [96] also set relatively narrow time constraints: Ostariophysi (125-149 Ma), Characiformes (68-100 Ma), and Siluriformes (73-83.5 Ma). Accordingly, their resulting estimates (Table 9) fit very well with the fossil records and little chance exists (<5%) that the MRCA of Otocephala is more than 152 Ma.

We recognize that the approach in Santini et al. [96] is superior in that it can lend more credence to the fossil record than the standard minimum-age constraints. Nevertheless, we did not provide upper (maximum) bounds for these nodes or manipulate two parameters of the truncated Cauchy distributions (= lognormal distributions in Santini et al. [96]) because insufficient information exists with which to specify meaningful prior distributions for most otophysan diversification times. Thus, the remarkable gaps between estimated ages from the two studies mostly reflect differences in priors on node ages (as the time constraints) rather than differences in software (MCMCTREE vs. BEAST) or data (mitogenomes vs. nuclear genes).

Some recent studies have argued that mitochondrial genes are likely to yield older node ages than nuclear genes. For example, Hurley et al. [100] investigated the relationships and divergence times of the basal actinopterygians (including teleosts) using four nuclear genes and whole mitogenomes. A relaxed molecular clock Bayesian analysis based on these two data sets provided node ages for the most recent common ancestor of teleosts with posterior means of 219 Ma (181-265 Ma) and 246 Ma (206-292 Ma) depending orthologues in the nuclear genes (only the former estimate shown in their figure 4) and 296 Ma (268-326 Ma) in the mitochondrial genome (data taken from their supplementary tables 3 and 7). Despite the overlap between these two results (95% credible intervals), Hurley et al. [100] concluded that the mitochondrial estimate of the most recent common ancestor of teleosts was 50-100 million years older than that of the nuclear genes. Based on this comparison, they further argued that the discrepancy between their nuclear and mitochondrial estimates may have been due to evolutionary rate differences between these two genomes. Note that their nuclear estimation did not include any species from the osteoglossomorphs (the putative most primitive teleosts; see Figure 3), which is likely to result in underestimation of the crown node age of teleosts with insufficient taxon sampling.

More recently, Brandley et al. [52] investigated intercontinental dispersal of *Plestiodon *(*Eumeces*) lizards based on analysis of a single mitochondrial gene (ND1) plus seven nuclear genes. They performed a relaxed molecular clock Bayesian divergence time estimation using unpartitioned and partitioned data sets across these genes and found that extreme saturation obscured the underlying rate of evolution in the mitochondrial gene, resulting in overestimation of the divergence times. Such overestimation in the mitochondrial gene was most pronounced in the unpartitioned data set and less so in the two partitioned data sets. Brandley et al. [52] agreed with Phillips [101], who demonstrated that genes or gene partitions that evolve at extremely high rates may accumulate so many hidden substitutions, making it difficult to estimate the underlying process that created the data. Although their arguments are convincing, comparisons made by Brandley et al. [52] were based on a single mitochondrial gene, including third codon positions. We used whole mitochondrial genomes with various substitution rates across genes [102], excluded quickly saturated third codon positions entirely, and partitioned the data sets.

Also the estimated ages may be too old simply because of the entire absence of a fossil record [100]. The absence of fossils, however, should not be taken as evidence of absence, as discussed extensively by Diogo [41]. In an article on the early radiation of teleosts, Arratia [42] stated "... we know almost nothing concerning the fossil record of most otophysans, of most living perciforms, atheriniforms, etc." Thus, to assume a lack of otophysan fossil record during the Mesozoic is natural if the group existed as stem groups during this period.

As Brown et al. [72] convincingly argued, although ample room for improvement exists on both sides of the "rock-clock" divide (e.g., accounting for ghost lineages in the fossil record and developing more realistic models of rate evolution for molecular genetic sequences), the consistent and conspicuous disagreement between these two sources of data more likely reflects a genuine difference between estimated ages of 1) stem group origins and 2) crown group morphological diversification, respectively.

### Comments on trans-Atlantic clades

Although this study was not intended to address questions regarding historical biogeography of the otophysans, divergence times of some of the transcontinental sister group relationships, particularly those between Africa and South America in Characiformes and Siluriformes, warrant brief comments regarding their origins.

Based on a hypothesis presented by Backup (unpublished thesis [103]), Lundberg [37] examined the implications of applying a strict vicariance scenario to the three transcontinental sister group pairs in Characiformes and noted that most of the diversification among characiforms would have occurred prior to the continental breakup (i.e., on Gondwana). Our timetree corroborates this hypothesis because all of our divergence time estimates of the three transcontinental pairs (Citharinoidei vs. Characoidei = 220 Ma; two alestids *Micralestes *+ *Phenacogrammus *vs. *Hydrolycus *= 162 Ma; *Hepsetus *vs. *Hoplias *= 160 Ma) fall well before the Gondwanan separation (100-120 Ma) [104]. Note, however, that recent discoveries of marine or brackish characiforms (*Santanichthys *[94] and *Sorbinicharax *[105]) from the Cretaceous challenge such a simple vicariance hypothesis [106].

Recently Lundberg et al. [107] examined the relationships of a highly distinct freshwater catfish, *Lacantunia enigmatica *(Lacantuniidae) from Chipas, Mexico, and found that *Lacantunia *was derived from within a multifamily clade of African freshwater catfishes ("Big Africa" in Sullivan et al. [21]; see Figures 3, 6). Based on a maximum-age constraint of 144 Ma for the stem of the siluriform lineage (= siluriform + gymnotiform node) and seven additional minimum-age constraints for the internal nodes, they estimated the divergence time of *Lacantunia *using a relaxed-molecular clock method implemented in MULTIDIVTIME [69] and r8s [108]. The resulting divergence estimates ranged from 83 to 87 Ma depending on the partitioning schemes with 95% credible intervals of 75-94 Ma. These ages fall after the separation of Africa and South America, and Lundberg et al. [107] proposed a new scenario based on an ancient intercontinental passage for explaining disjunct distributions of relictually endemic *Lacantunia *and its African sister clade. Also note that they acknowledge that their choice of 144 Ma as the maximum-age constraint is arbitrary, but conservatively informed by the fossil record of actinopterygians. Although we did not include *Lacantunia *in our analyses, the onset of diversification of the Big African clade is estimated to be 129 Ma (113-144 Ma) and divergence of *Lacantunia *from its stem should be much older. Thus our estimated ages did not require an ancient intercontinental passage as hypothesized by Lundberg et al. [107].

### History of diversification

ML reconstruction of the ancestral habitats on the timetree suggests that a shift from character state 0 (saltwater) to 1 (freshwater) occurred between the crown node of Ostariophysi (*P*_0 _= 0.859; *P*_1 _= 0.142) and that of Otophysi (*P*_0 _= 0.055; *P*_1 _= 0.945), corresponding to the end-Permian (263-249 Ma) (Figure 6). Moreover, a similar habitat shift was also recovered in Gonorynchiformes, a sister clade of Otophysi (Figure 6). Based on these ancestral habitat reconstructions and the divergence time estimates with reference to Earth's history, we identified the following five global turning points in the history of otophysan diversification. Note, however, that the true ancestral habitats and dynamics of diversification cannot be inferred from time-calibrated phylogenies of the extant lineages alone [109]; one need to include the fossil record to fully understand the ancestral habitats and diversity dynamics of the otophysans.

First, the common ancestor of Otophysi presumably entered freshwater around 263-249 Ma. This period corresponds to the end-Permian when the largest mass extinction in Earth's history occurred, wiping out 96% of all species [110]. The end-Permian mass extinction has been associated with a massive release of carbon gases into the atmosphere, causing a global greenhouse effect and abrupt climate warming [111]. During the same time, the oceans are believed to have become anoxic worldwide [112,113] and to have contained free hydrogen sulfide [114]. These environmental perturbations greatly altered the marine ecosystems [115] and may have driven fish extinctions. Note that freshwater contains much higher levels of dissolved oxygen than saltwater for a given atmospheric concentration, and this difference can affect the biology of the organisms [116]. The possible habitat shift (from salt- to freshwater) in the common ancestor of Otophysi should have a causal relationship with the survival of this lineage across the end-Permian. To our knowledge, however, no comparable examples of habitat shift in other groups of organisms exist that independently support this evolutionary scenario.

Second, the five major otophysan lineages (corresponding to common ancestors of the present-day orders or suborders) have successively originated from an ancestral lineage of the otophysans in a short time window during the Triassic (251-200 Ma). During this period all continents remained united as the supercontinent Pangaea, with a climate characterized by globally warm temperatures, extreme seasonality, and high aridity over much of the inland region [117]. However, the climate was more moderate around the edges of the supercontinent. The regions around Pangaea had sufficient rainfall to produce vigorous forests along riverbanks [118], which possibly facilitated stepwise divergences of the five lineages on Pangaea. Note that the Pangaea was cut almost in half along the east-west axis by a huge embayment called the Tethys Seaway (Figure 6). Over time, as the Tethys Seaway expanded, it may have led to the first vicariant divergence between the common ancestors of Cypriniformes and Characiphysi as suggested by Saitoh *et al. *[4,12].

Third, these five major otophysan lineages survived the end-Triassic mass extinction, which was one of the five largest extinctions in Earth's history in which 80% of all species became extinct [117]. Widespread magmatic activity of the Central Atlantic Magmatic Province (CAMP) has been suggested to have caused this catastrophic event, and repeated release of SO_2 _gas, heavy metal emissions, and darkening were the main environmental stressors [119]. Relatively long stem branches from the common ancestors of the five major lineages across the Triassic-Jurassic boundary suggest profound effects of the mass extinction and associated environmental perturbations on the patterns of otophysan diversification.

Fourth, extant otophysan lineages began to diversify in each of the five clades during the Jurassic and through the early Cretaceous (200-100 Ma) (Figure 6), providing a framework for the modern otophysan diversity. The onset of diversification depends on the clade (193-160 Ma), although all fell in the Jurassic (200-146 Ma). Note that familial diversification of the South American gymnotiforms, characoids, and loricaroids was recovered much earlier than that of cypriniforms, citharinoids and siluroids (Figure 6). The Jurassic was a time of particularly swift change due to the Pangaean breakup and the resulting development of new oceans and a gentle tropical climate over the formerly arid interiors of the supercontinent [118]. Such environmental changes likely yielded numerous novel habitats, facilitating otophysan diversification throughout the Mesozoic until about 100 Ma.

Finally these modern otophysan lineages established during the Mesozoic survived the third mass extinction event at the end of the Cretaceous (Figure 6). Such "mass survival" of the modern lineages across the Cretaceous-Paleogene boundary has been noted for birds and mammals based on molecular evidence [120].

The present timetree suggests a Pangaean origin and Mesozoic radiations of the modern otophysans. This evolutionary scenario is in good agreement with recent biogeographic inferences. For example Diogo [41] broadly surveyed the higher-level phylogeny, biogeographic distribution, physiology, and ecology of catfishes, and suggested that these fishes originated at a time when some Pangaean connections still existed between Laurasia and Gondwana. In addition, Briggs [33] examined the phylogeny and geographic distribution patterns of ostariophysan fishes and proposed the Late Jurassic (160-150 Ma) origins of the cypriniforms and siluriforms, which are highly congruent with the estimated node ages for the most recent common ancestor of Cypriniformes (160 Ma) and Siluriformes (180 Ma) in this study.

Of course the evolutionary scenario presented here just represents a testable hypothesis and should be viewed with caution because the fossil record provides no direct evidence. Although Mesozoic freshwater deposits are geographically and stratigraphically patchy and the freshwater fishes from most of these deposits have not been fully documented across all continents [121-123], available information suggests that major components of the freshwater fishes were basal actinopterygians (non-teleost ray-finned fishes), chondrichthyans (sharks, rays, chimaeras) and sarcopterygians (lungfishes and coelacanths) [121-124], and few freshwater teleosts (e.g., the Late Triassic *Jiangilichthys*) are referable to extinct "pholidophoriforms," phylogenetically located outside the modern teleosts [125]. Moreover the fossil record suggests that the modern teleostean lineages did not diversify until the Late Jurassic about 150 Ma [125]. Thus, acceptance of the present evolutionary scenario requires the origin and survival of the ghost lineage from the Triassic through Late Jurassic for over 100 million years.

## Conclusions

The timetree presented here indicates that survival of the ancestral lineages through the two consecutive mass extinctions on Pangaea and subsequent radiations during the Jurassic and through the early Cretaceous shaped the modern familial diversity of otophysans. The Pangaean origin and Mesozoic radiations of the modern otophysans are consistent with recent arguments based on biogeographic inferences [40,41] and molecular divergence time estimates [37,40]. No fossil otophysan, however, has been recorded before the Albian, the early Cretaceous 100-112 Ma [42], creating an over 100 million year time span without fossil evidence. This extremely large ghost range partially reflects a genuine difference between the estimated ages of stem group origin (molecular divergence time) and crown group morphological diversification (fossil divergence time) [72]; the ghost range, however, may be filled with future discoveries of older fossils that can be used as more reasonable time constraints as well as with the development of more realistic models that accurately capture the divergence rates of molecular sequences.

## Authors' contributions

MNa, MM, KM and MNi designed this study. MNa and KS mainly collected the specimens and carried out the molecular work. MNa and MM analyzed the data. MM drafted the original manuscript and MNa, KM, KS and MNi contributed to its improvement. All authors read and approved the final manuscript.

## Supplementary Material

Additional file 1 A cladogram showing all nodes with < 50% BSPs in Figure 3 collapsed to polytomy. Click here for file

Additional file 2 **Summary of MCMC samples for node ages (means and 95% credible intervals) in the independent-rates (IR) and autocorrelated-rates (AR) analyses using MCMCTREE-HS **[63]. Results from the two data sets (12_n_RT_n _and 123_a_) are shown separately. For node numbers, see tree at the end of this file. Click here for file

Additional file 3 **Timetree derived from the Bayesian relaxed-molecular clock method (non-ostariophysan portions also shown)**. Upper (maximum) and lower (minimum) time constraints used in this study are shown by arrowheads with corresponding nodes connected by dotted lines. All marine species are indicated by asterisks. Click here for file

## References

[B1] Dudgeon D Arthington AH Gessner MO Kawabata ZI Knowler DJ Lévéque C Naiman RJ Prieur-Richard AH Soto D Stiassny MLJ Freshwater biodiversity: importance, threats, status and conservation challenges Biol Rev 2005 81 02 163 182 16336747 10.1017/S1464793105006950

[B2] Schindler DE Fish extinctions and ecosystem functioning in tropical ecosystems Proc Natl Acad Sci USA 2007 104 14 5707 5708 10.1073/pnas.0700426104 17392429 PMC1851553

[B3] Nelson JS Fishes of the world 2006 4 Hoboken, NJ: John Wiley & Sons

[B4] Saitoh K Miya M Inoue JG Ishiguro NB Nishida M Mitochondrial genomics of ostariophysan fishes: Perspectives on phylogeny and biogeography J Mol Evol 2003 56 4 464 472 10.1007/s00239-002-2417-y 12664166

[B5] Poulsen JY Møller PDR Lavoué S Knudsen SW Nishida M Miya M Higher and lower-level relationships of the deep-sea fish order Alepocephaliformes (Teleostei: Otocephala) inferred from whole mitogenome sequences Biol J Linn Soc 2009 98 4 923 936 10.1111/j.1095-8312.2009.01323.x

[B6] Lavoué S Miya M Inoue JG Saitoh K Ishiguro NB Nishida M Molecular systematics of the gonorynchiform fishes (Teleostei) based on whole mitogenome sequences: Implications for higher-level relationships within the Otocephala Mol Phylogenet Evol 2005 37 1 165 177 10.1016/j.ympev.2005.03.024 15890536

[B7] Lavoué S Miya M Poulsen JY Møller PR Nishida M Monophyly, phylogenetic position and inter-familial relationships of the Alepocephaliformes (Teleostei) based on whole mitogenome sequences Mol Phylogenet Evol 2008 47 3 1111 1121 10.1016/j.ympev.2007.12.002 18262798

[B8] Lecointre G Grande T, Poyato-Ariza FJ, Diogo R Gonorynchiformes in the teleostean phylogeny: molecules and morphology used to investigate interrelationships of the Ostariophysi Gonorynchiformes and ostariophysan relationships: a comprehensive review 2010 Enfield, NH: Science Publishers

[B9] Miya M Satoh TP Nishida M The phylogenetic position of toadfishes (order Batrachoidiformes) in the higher ray-finned fish as inferred from partitioned Bayesian analysis of 102 whole mitochondrial genome sequences Biol J Linn Soc 2005 85 3 289 306 10.1111/j.1095-8312.2005.00483.x

[B10] Miya M Takeshima H Endo H Ishiguro NB Inoue JG Mukai T Satoh TP Yamaguchi M Kawaguchi A Mabuchi K Major patterns of higher teleostean phylogenies: a new perspective based on 100 complete mitochondrial DNA sequences Mol Phylogenet Evol 2003 26 1 121 138 10.1016/S1055-7903(02)00332-9 12470944

[B11] Saitoh K Sado T Mayden RL Hanzawa N Nakamura K Nishida M Miya M Mitogenomic evolution and interrelationships of the Cypriniformes (Actinopterygii: Ostariophysi): The first evidence toward resolution of higher-level relationships of the World's largest freshwater fish clade based on 59 whole mitogenome sequences J Mol Evol 2006 63 6 826 841 10.1007/s00239-005-0293-y 17086453

[B12] Saitoh K Sado T Doosey MH Bart HLJ Inoue JG Nishida M Mayden RL Nishida M Miya M Evidence from mitochondrial genomics supports the lower Mesozoic of South Asia as the time and place of basal divergence of cypriniform fishes (Actinopterygii: Ostariophysi) Zool J Linn Soc 2011 161 633 662 10.1111/j.1096-3642.2010.00651.x

[B13] Mayden RL Tang KL Wood RM Chen W-J Agnew MK Conway KW Yang L Simons AM Bart HL Harris PM Inferring the Tree of Life of the order Cypriniformes, the earth's most diverse clade of freshwater fishes: implications of varied taxon and character sampling J Systemat Evol 2008 46 3 424 438

[B14] Chen WJ Miya M Saitoh K Mayden RL Phylogenetic utility of two existing and four novel nuclear gene loci in reconstructing Tree of Life of ray-finned fishes: The order Cypriniformes (Ostariophysi) as a case study Gene 2008 423 2 125 134 10.1016/j.gene.2008.07.016 18703121

[B15] He S Gu X Mayden RL Chen W-J Conway KW Chen Y Phylogenetic position of the enigmatic genus *Psilorhynchus *(Ostariophysi: Cypriniformes): Evidence from the mitochondrial genome Mol Phylogenet Evol 2008 47 1 419 425 10.1016/j.ympev.2007.10.012 18053751

[B16] Mayden RL Chen WJ Bart HL Doosey MH Simons AM Tang KL Wood RM Agnew MK Yang L Hirt MV Reconstructing the phylogenetic relationships of the earth's most diverse clade of freshwater fishes --order Cypriniformes (Actinopterygii: Ostariophysi): A case study using multiple nuclear loci and the mitochondrial genome Mol Phylogenet Evol 2009 51 3 500 514 10.1016/j.ympev.2008.12.015 19141325

[B17] Mayden RL Chen WJ The world's smallest vertebrate species of the Genus *Paedocypris*: A new family of freshwater fishes and the sister group to the world's most diverse clade of freshwater fishes (Teleostei: Cypriniformes) Mol Phylogenet Evol 2010 57 1 152 175 10.1016/j.ympev.2010.04.008 20398777

[B18] Chen W-J Mayden RL Molecular systematics of the Cyprinoidea (Teleostei: Cypriniformes), the World's largest clade of freshwater fishes: further evidence from six nuclear genes Mol Phylogenet Evol 2009 52 544 549 10.1016/j.ympev.2009.01.006 19489125

[B19] Ortí G Meyer A The radiation of characiform fishes and the limits of resolution of mitochondrial ribosomal DNA sequences Syst Biol 1997 46 1 75 100 10.1093/sysbio/46.1.75 11975355

[B20] Calcagnotto D Schaefer SA Desalle R Relationships among characiform fishes inferred from analysis of nuclear and mitochondrial gene sequences Mol Phylogenet Evol 2005 36 1 135 153 10.1016/j.ympev.2005.01.004 15904862

[B21] Sullivan J Lundberg J Hardman M A phylogenetic analysis of the major groups of catfishes (Teleostei: Siluriformes) using rag1 and rag2 nuclear gene sequences Mol Phylogenet Evol 2006 41 3 636 662 10.1016/j.ympev.2006.05.044 16876440

[B22] Hardman M The phylogenetic relationships among non-diplomystid catfishes as inferred from mitochondrial cytochrome b sequences; the search for the ictalurid sister taxon (Otophysi: Siluriformes) Mol Phylogenet Evol 2005 37 3 700 720 10.1016/j.ympev.2005.04.029 16054398

[B23] Alves-Gomes JA Orti G Haygood M Heiligenberg W Meyer A Phylogenetic analysis of the South American electric fishes (order Gymnotiformes) and the evolution of their electrogenic system: a synthesis based on morphology, electrophysiology, and mitochondrial sequence data Mol Biol Evol 1995 12 298 318 7700155 10.1093/oxfordjournals.molbev.a040204

[B24] Alves-Gomes JA Grande T, Poyato-Ariza FJ, Diogo R The mitochondrial phylogeny of the South American electric fish (Gymnotiformes) and an alternative hypothesis for the otophysan historical biogeography Gonorynchiformes and ostriophysan relationships: A comprehensive review 2010 Enfield, NH: Science Publishers 517 565

[B25] Hennig W Phylogenetic systematics 1996 Urbana, IL: University of Illinois Press

[B26] Wiley EO Phylogenetics: the theory and practice of phylogenetic systematics 1981 New York: Wiley & Sons

[B27] Fink SV Fink WL Interrelationships of the ostariophysan fishes (Teleostei) Zool J Linn Soc 1981 72 4 297 353 10.1111/j.1096-3642.1981.tb01575.x

[B28] Dimmick WW Larson A A molecular and morphological perspective on the phylogenetic relationships of the otophysan fishes Mol Phylogenet Evol 1996 6 1 120 133 10.1006/mpev.1996.0064 8812312

[B29] Ortí G Meyer A Molecular evolution of ependymin and the phylogenetic resolution of early divergences among euteleost fishes Mol Biol Evol 1996 13 4 556 573 8882499 10.1093/oxfordjournals.molbev.a025616

[B30] Peng Z He S Wang J Wang W Diogo R Mitochondrial molecular clocks and the origin of the major otocephalan clades (Pisces: Teleostei): a new insight Gene 2006 370 113 124 16476526 10.1016/j.gene.2005.11.024

[B31] Li C Lu G Orti G Optimal data partitioning and a test case for ray-finned fishes (Actinopterygii) based on ten nuclear loci Syst Biol 2008 57 4 519 539 10.1080/10635150802206883 18622808

[B32] Novacek MJ Marshall LG Early biogeographic history of ostariophysan fishes Copeia 1976 1976 1 1 12 10.2307/1443767

[B33] Briggs JC The biogeography of otophysan fishes (Ostariophysi: Otophysi): a new appraisal J Biogeogr 2005 32 2 287 294 10.1111/j.1365-2699.2004.01170.x

[B34] Briggs JC Ostariophysan zoogeography: an alternative hypothesis Copeia 1979 1979 111 118 10.2307/1443735

[B35] Gayet M Consideration sur la phylogenie et la paleobiographie des Ostariophysaries Geobios 1982 6 39 52

[B36] Banarescu P Zoogeography of fresh waters 1990 1 Wiesbaden, Germany: AULA-Verlag

[B37] Lundberg JG Goldblatt P African-South American freshwater clades and continental drift: problems with a paradigm Biological relationships between Africa and South America 1993 New Haven: Yale University Press 156 199

[B38] Fara E Gayet M Taverne L Grande T, Poyato-Ariza FJ, Diogo R The Fossil record of Gonorynchiformes Gonorynchiformes and ostariophysan relationships: A comprehensive review 2010 Enfield, NH: Science Publishers 173 226

[B39] Malabarba MC Malabarba LR Nelson JS, Schultze H-P, Wilson MVH Biogeography of Characiformes: an evaluation of the available information of fossil and extant taxa Origin and Phylogenetic Interrelationships of Teleosts 2010 München: Verlag Dr. Friedrich Pfeil

[B40] Conway KW Hirt MV Yang L Mayden RL Simons AM Nelson JS, Schultze H-P, Wilson MVH Cypriniformes: systematics and paleontology Origin and phylogenetic interrelationships of teleosts 2010 München: Verlag Dr. Friedrich Pfeil

[B41] Diogo R Phylogeny, origin and biogeography of catfishes: support for a Pangean origin of 'modern teleosts' and reexamination of some Mesozoic Pangean connections between the Gondwanan and Laurasian supercontinents Anim Biol 2004 54 4 331 351 10.1163/1570756042729546

[B42] Arratia G Arratia G, Tintori A Mesozoic halecostomes and the early radiation of teleosts Mesozoic fishes 3 2004 München, Germany: Verlag Dr. Friedrich Pfeil 279 315

[B43] Azuma Y Kumazawa Y Miya M Mabuchi K Nishida M Mitogenomic evaluation of the historical biogeography of cichlids toward reliable dating of teleostean divergences BMC Evol Biol 2008 8 1 215 10.1186/1471-2148-8-215 18651942 PMC2496912

[B44] Asahida T Kobayashi T Saitoh K Nakayama I Tissue preservation and total DNA extraction from fish stored at ambient temperature using buffers containing high concentration of urea Fish Sci 1996 62 727 730

[B45] Miya M Nishida M Organization of the mitochondrial genome of a deep-sea fish, *Gonostoma gracile *(Teleostei: Stomiiformes): first example of transfer RNA gene rearrangements in bony fishes Mar Biotechnol 1999 1 5 416 426 10.1007/PL00011798 10525676

[B46] Cheng S Chang SY Gravitt P Respess R Long PCR Nature 1994 369 6482 684 645 10.1038/369684a0 8208299

[B47] Katoh K Toh H Recent developments in the MAFFT multiple sequence alignment program Brief Bioinformatics 2008 9 4 286 298 10.1093/bib/bbn013 18372315

[B48] Maddison WP Maddison DR MacClade Version 3 2000 Sunderland: Sinauer Associates

[B49] Miya M Nishida M Use of mitogenomic information in teleostean molecular phylogenetics: a tree-based exploration under the maximum-parsimony optimality criterion Mol Phylogenet Evol 2000 17 3 437 455 10.1006/mpev.2000.0839 11133198

[B50] Broughton RE Nelson JS, Shultze H-S, Wilson MVH Phylogeny of teleosts based on mitochondrial genome sequences Origin and phylogenetic interrelationships of teleosts 2010 München, Germany: Verlag Dr. Friedrich Pfeil 61 76

[B51] Benton MJ Ayala FJ Dating the Tree of Life Science 2003 300 5626 1698 1700 10.1126/science.1077795 12805535

[B52] Brandley MC Wang Y Guo X de Oca ANM Fería-Ortíz M Hikida T Ota H Accommodating high rates of evolution in molecular dating methods: an example using inter-continental dispersal of *Plestiodon *(Eumeces) lizards Syst Biol 2011 60 1 3 15 10.1093/sysbio/syq045 20952756

[B53] Stamatakis A RAxML-VI-HPC: maximum likelihood-based phylogenetic analyses with thousands of taxa and mixed models Bioinformatics 2006 22 21 2688 2690 10.1093/bioinformatics/btl446 16928733

[B54] Yang Z Estimating the pattern of nucleotide substitution J Mol Evol 1994 39 1 105 111 8064867 10.1007/BF00178256

[B55] Posada D Crandall KA MODELTEST: testing the model of DNA substitution Bioinformatics 1998 14 9 817 818 10.1093/bioinformatics/14.9.817 9918953

[B56] Adachi J Hasegawa M Model of amino acid substitution in proteins encoded by mitochondrial DNA J Mol Evol 1996 42 4 459 468 10.1007/BF02498640 8642615

[B57] Shimodaira H Hasegawa M CONSEL: for assessing the confidence of phylogenetic tree selection Bioinformatics 2001 17 12 1246 1247 10.1093/bioinformatics/17.12.1246 11751242

[B58] Shimodaira H An approximately unbiased test of phylogenetic tree selection Syst Biol 2002 51 3 492 508 10.1080/10635150290069913 12079646

[B59] Felsenstein J Confidence limits on phylogenies: an approach using the bootstrap Evolution 1985 39 4 783 791 10.2307/2408678 28561359

[B60] Kishino H Hasegawa M Evaluation of the maximum likelihood estimate of the evolutionary tree topologies from DNA sequence data, and the branching order in hominoidea J Mol Evol 1989 29 2 170 179 10.1007/BF02100115 2509717

[B61] Shimodaira H Hasegawa M Multiple comparisons of log-likelihoods with applications to phylogenetic inference Mol Biol Evol 1999 16 1114 1116

[B62] Kawahara R Miya M Mabuchi K Near T Nishida M Stickleback phylogenies resolved: Evidence from mitochondrial genomes and 11 nuclear genes Mol Phylogenet Evol 2009 50 1 401 404 19027080 10.1016/j.ympev.2008.10.014

[B63] Yang Z PAML 4: phylogenetic analysis by maximum likelihood Mol Biol Evol 2007 24 8 1586 1591 10.1093/molbev/msm088 17483113

[B64] Drummond AJ Rambaut A BEAST: Bayesian evolutionary analysis by sampling trees BMC Evol Biol 2007 7 214 10.1186/1471-2148-7-214 17996036 PMC2247476

[B65] Rannala B Yang Z Inferring speciation times under an episodic molecular clock Syst Biol 2007 56 3 453 466 10.1080/10635150701420643 17558967

[B66] Zhong B Yonezawa T Zhong Y Hasegawa M Episodic evolution and adaptation of chloroplast genomes in ancestral grasses PLoS ONE 2009 4 4 e5297 10.1371/journal.pone.0005297 19390686 PMC2669172

[B67] Yang Z Rannala B Bayesian phylogenetic inference using DNA sequences: a Markov chain Monte Carlo method Mol Biol Evol 1997 14 7 717 724 9214744 10.1093/oxfordjournals.molbev.a025811

[B68] Setiamarga DHE Miya M Inoue JG Ishiguro NB Mabuchi K Nishida M Divergence time of the two regional medaka populations in Japan as a new time scale for comparative genomics of vertebrates Biol Lett 2009 5 5 81 86 19586967 10.1098/rsbl.2009.0419 PMC2827986

[B69] Thorne JL Kishino H Divergence time and evolutionary rate estimation with multilocus data Syst Biol 2002 51 5 689 702 10.1080/10635150290102456 12396584

[B70] Benton MJ Donoghue PCJ Paleontological evidence to date the Tree of Life Mol Biol Evol 2006 24 1 26 53 10.1093/molbev/msl150 17047029

[B71] Yang Z Rannala B Bayesian estimation of species divergence times under a molecular clock using multiple fossil calibrations with soft bounds Mol Biol Evol 2006 23 1 212 226 16177230 10.1093/molbev/msj024

[B72] Brown JW Rest JS García-Moreno J Sorenson MD Mindell DP Strong mitochondrial DNA support for a Cretaceous origin of modern avian lineages BMC Biol 2008 6 6 18226223 10.1186/1741-7007-6-6 PMC2267772

[B73] Mesquite: a modular system for evolutionary analysis Ver. 2.73 http://mesquiteproject.org

[B74] Page M The maximum likelihood approach to reconstructing ancestral character states of discrete characters on phylogenies Syst Biol 1999 48 3 612 622 10.1080/106351599260184

[B75] Schluter D Price T Mooers A Ludwig D Likelihood of ancestor states in adaptive radiation Evolution 1997 51 6 1699 1711 10.2307/2410994 28565128

[B76] Lewis PO A likelihood approach to estimating phylogeny from discrete morphological character data Syst Biol 2001 50 6 913 925 10.1080/106351501753462876 12116640

[B77] Lartillot N Philippe H A Bayesian mixture model for across-site heterogeneities in the amino-acid replacement process Mol Biol Evol 2004 21 6 1095 1109 10.1093/molbev/msh112 15014145

[B78] Lartillot N Lepage T Blanquart S PhyloBayes 3: a Bayesian software package for phylogenetic reconstruction and molecular dating Bioinformatics 2009 25 17 2286 2288 10.1093/bioinformatics/btp368 19535536

[B79] Buckup PA Malabarba LR, Reis RE, Vari RP, Lucena ZMS, Lucena CAS Relationships of the Characidiinae and phylogeny of characiform fishes (Teleostei: Ostariophysi) Phylogeny and classification of neotropical fishes 1998 Porto Alegre, Brasil: Edipucrs 123 144

[B80] Fink SV Fink WL Stiassny MLJ, Parenti LR, Johnson GD Interrelationships of ostariophysan fishes (Teleostei) Interrelationships of fishes 1996 San Diego: Academic Press 209 249

[B81] Dahdul WM Grande T, Poyato-Ariza FJ, Diogo R Review of the phylogenetic relationships and fossil record of Characiformes Gonorynchiformes and ostriophysan relationships: A comprehensive review 2010 Enfield, New Hampshire: Science Publishers 441 464

[B82] Vari RP Anatomy, relationships and classification of the families Citharinidae and Distichodontidae (Pisces, Characoidea) Bull Brit Mus (Nat Hist) Zool Ser 1979 36 261 344

[B83] Ortí G Petry P Porto JIR Jégu M Meyer A Patterns of nucleotide change in mitochondrial ribosomal RNA genes and the phylogeny of piranhas J Mol Evol 1996 42 2 169 182 10.1007/BF02198843 8919869

[B84] Grande L Redescription of *Hypsidoris farsonensis *(Teleostei: Siluriformes), with a reassessment of its phylogenetic relationships J Vert Paleontol 1987 7 24 54 10.1080/02724634.1987.10011636

[B85] Mo T Anatomy, relationships and systematics of the Bagridae (Teleostei: Siluroidei) with a hypothesis of siluroid phylogeny Theses Zoology 1991 17 1 216

[B86] Arratia G Development and variation of the suspensorium of primitive catfishes (Teleostei: Ostariophysi) and their phylogenetic relationships Bonn Zool Monogr 1992 32 1 148

[B87] De Pinna MCC Malabarba LR, Reis RE, Vari RP, Lucena ZM, Lucena CAS Phylogenetic relationships of Neotropical Siluriformes: History, overview and synthesis of hypotheses Phylogeny and Classification of Neotropical Fishes 1998 Porto Alegre, Brasil: Edipucrs 279 330

[B88] He S Gayet M Meunier FJ Phylogeny of the Amphiliidae (Teleostei: Siluriformes) Ann Sci Natur 1999 20 4 117 146

[B89] Diogo R Peng Z Grande T, Poyato-Ariza FJ, Diogo R State of the art of siluriform higher-level phylogeny Gonorynchiformes and ostriophysan relationships: A comprehensive review 2010 Enfield, New Hampshire: Science Publishers 465 515

[B90] Inoue JG Miya M Venkatesh B Nishida M The mitochondrial genome of Indonesian coelacanth *Latimeria menadoensis *(Sarcopterygii: Coelacanthiformes) and divergence time estimation between the two coelacanths Gene 2005 349 227 235 15777665 10.1016/j.gene.2005.01.008

[B91] Yamanoue Y Miya M Inoue JG Matsuura K Nishida M The mitochondrial genome of spotted green pufferfish *Tetraodon nigroviridis *(Teleostei: Tetraodontiformes) and divergence time estimation among model organisms in fishes Genes Genet Syst 2006 81 1 29 39 10.1266/ggs.81.29 16607039

[B92] Inoue JG Kumazawa Y Miya M Nishida M The historical biogeography of the freshwater knifefishes using mitogenomic approaches: a Mesozoic origin of the Asian notopterids (Actinopterygii: Osteoglossomorpha) Mol Phylogenet Evol 2009 51 3 486 499 10.1016/j.ympev.2009.01.020 19444960

[B93] Arratia G Basal teleosts and teleostean phylogeny Palaeo-Ichthyology 1997 7 5 168

[B94] Filleul A Maisey JG Redescription of *Santanichthys diasii *(Otophysi, Characiformes) from the Albian of the Santana Formation and comments on its implications for otophysan relationships Am Mus Novit 2004 3455 1 21 10.1206/0003-0082(2004)455<0001:ROSDOC>2.0.CO;2

[B95] Kumazawa Y Azuma Y Nishida M Tempo of mitochondrial gene evolution: Can mitochondrial DNA be used to date old divergences? Endocytobiosis Cell Research 2004 15 136 142

[B96] Santini F Harmon LJ Carnevale G Alfaro ME Did genome duplication drive the origin of teleosts? A comparative study of diversification in ray-finned fishes BMC Evol Biol 2009 9 1 194 10.1186/1471-2148-9-194 19664233 PMC2743667

[B97] Donoghue PCJ Benton MJ Rocks and clocks: calibrating the Tree of Life using fossils and molecules Trends Ecol Evol 2007 22 8 424 431 10.1016/j.tree.2007.05.005 17573149

[B98] Reisz RR Müller J Molecular timescales and the fossil record: a paleontological perspective Trends Genet 2004 20 237 241 10.1016/j.tig.2004.03.007 15109777

[B99] Müller J Reisz RR Four well-constrained calibration points from the vertebrate fossil record for molecular clock estimates Bioessays 2005 27 1069 1075 10.1002/bies.20286 16163732

[B100] Hurley I Mueller R Dunn K Schmidt E Friedman M Ho R Prince V Yang Z Thomas M Coates M A new time-scale for ray-finned fish evolution Proc R Soc B 2007 274 1609 489 498 10.1098/rspb.2006.3749 17476768 PMC1766393

[B101] Phillips MJ Branch-length estimation bias misleads molecular dating for a vertebrate mitochondrial phylogeny Gene 2009 441 1-2 132 140 10.1016/j.gene.2008.08.017 18809474

[B102] Miya M Saitoh K Wood R Nishida M Mayden RL New primers for amplifying and sequencing the mitochondrial ND4/ND5 gene region of the Cypriniformes (Actinopterygii: Ostariophysi) Ichthyol Res 2006 53 1 75 81 10.1007/s10228-005-0303-5

[B103] Buckup PA The Characidiinae: a phylogenetic study of the South American darters and their relationships with other characiform fishes Ph.D. thesis 1991 Ann Harbor: The University of Michigan

[B104] Storey BC The role of mantle plumes in continental breakup: case histories from Gondwanaland Nature 1995 377 301 308 10.1038/377301a0

[B105] Taverne L Les poissons crétacés de Nardò. 16° *Sorbinicharax verraesi *gen. sp. nov. (Teleostei, Ostariophysi, Otophysi, Characiformes) Bollettino del Museo Civico di Storia Naturale di Verona 2003 27 29 45

[B106] Otero O Valentin X Garcia G Cavin L, Longbottom A, Richter M Cretaceous characiform fishes (Teleostei: Ostariophysi) from Northern Tethys: description of new material from the Maastrichtian of Provence (Southern France) and palaeobiogeographic implications Fishes and the break-up of Pangaea 2008 295 London: Geological Society, London, Special Publications 155 164

[B107] Lundberg JG Sullivan JP Rodiles-Hernández R Hendrickson DA Discovery of African roots for the Mesoamerican Chiapas catfish, Lacantunia enigmatica, requires an ancient intercontinental passage Proc Acad Nat Sci Phila 2007 156 1 39 53 10.1635/0097-3157(2007)156[39:DOARFT]2.0.CO;2

[B108] Sanderson MJ r8s ver. 1.70 2004

[B109] Quental TB Marshall CR Diversity dynamics: molecular phylogenies need the fossil record Trends Ecol Evol 2010 25 8 434 441 10.1016/j.tree.2010.05.002 20646780

[B110] Benton MJ Vertebrate palaeontology 2005 3 Malden, MA: Blackwell

[B111] Benton MJ Tverdokhlebov VP Surkov MV Ecosystem remodeling among vertebrates at the Permian-Triassic boundary in Russia Nature 2004 432 7013 97 100 10.1038/nature02950 15525988

[B112] Isozaki Y Permo-Triassic boundary superanoxia and stratified superocean: records from lost deep sea Science 1997 276 5310 235 238 10.1126/science.276.5310.235 9092467

[B113] Sperling EA Ingle JC Jr A Permian-Triassic boundary section at Quinn River Crossing, northwestern Nevada, and implications for the cause of the Early Triassic chert gap on the western Pangean margin Bull Geol Soc Am 2006 118 5-6 733 746 10.1130/B25803.1

[B114] Grice K Cao C Love GD B ttcher ME Twitchett RJ Grosjean E Summons RE Turgeon SC Dunning W Jin Y Photic zone euxinia during the Permian-Triassic superanoxic event Science 2005 307 5710 706 709 10.1126/science.1104323 15661975

[B115] Jin YG Wang Y Wang W Shang QH Cao CQ Erwin DH Pattern of marine mass extinction near the Permian-Triassic boundary in South China Science 2000 289 5478 432 436 10.1126/science.289.5478.432 10903200

[B116] Chapelle G Peck LS Polar gigantism dictated by oxygen availability Nature 1999 399 6732 114 115

[B117] Huynh TT Poulsen CJ Rising atmospheric CO_2 _as a possible trigger for the end-Triassic mass extinction Palaeogeogr, Palaeoclimatol, Palaeoecol 2005 217 3-4 223 242 10.1016/j.palaeo.2004.12.004

[B118] Dixon D Jenkins I Moody RTJ Zhuravlev AY Atlas of the evolving earth. Volume 2. From the Devonian to the Cretaceous 2001 New York: Macmillan Reference USA

[B119] Guex J Bartolini A Atudorei V Taylor D High-resolution ammonite and carbon isotope stratigraphy across the Triassic-Jurassic boundary at New York Canyon (Nevada) Earth Planet Sci Lett 2004 225 1-2 29 41 10.1016/j.epsl.2004.06.006

[B120] Cooper A Penny D Mass survival of birds across the Cretaceous-Tertiary boundary: molecular evidence Science 1997 275 1109 1113 10.1126/science.275.5303.1109 9027308

[B121] López-Arbarello A Arratia G, Tintori A The record of Mesozoic fishes from Gondwana (excluding India and Madagascar) Mesozoic fishes 3 2004 München, Germany: Verlag Dr. Friedrich Pfeil 597 624

[B122] Wilson MVH Bruner JC Arratia G Tintori A Mesozoic fish assemblages of North America Mesozoic fishes 3 2004 München, Germany: Verlag Dr. Friedrich Pfeil 575 595

[B123] Chang MM Miao D Arratia G Tintori A Arratia G, Tintori A An overview of Mesozoic fishes in Asia Mesozoic fishes 3 2004 München, Germany: Verlag Dr. Friedrich Pfeil 535 563

[B124] Milner ARC Kirkland JI Birthisel TA The geographic distribution and biostratigraphy of Late Triassic-Early Jurassic freshwater fish faunas of the southwestern United States New Mexico Museum of Natural History and Science Bulletin 2006 37 522 529

[B125] Arratia G Scasso R Kiessling W Late Jurassic fishes from Longing Gap, Antarctic Peninsula J Vert Paleontol 2004 24 1 41 55 10.1671/1952-4

[B126] Smith AG Smith DG Funnell BM Atlas of Mesozoic and Cenozoic coastlines 2004 Cambridge, UK: Cambridge University Press

[B127] Ziegler A Eshel G Rees PMA Rothfus T Rowley D Sunderlin D Tracing the tropics across land and sea: Permian to present Lethaia 2003 36 3 227 254 10.1080/00241160310004657

[B128] Janvier P Early vertebrates 1996 Oxford, UK: Oxford University Press

[B129] Zhu M Yu X Wang W Zhao W Jia L A primitive fish provides key characters bearing on deep osteichthyan phylogeny Nature 2006 441 7089 77 80 10.1038/nature04563 16672968

[B130] Patterson C Benton MJ Osteichthyes: Teleostei The fossil record 2 1993 London, UK: Chapman & Hall 621 656

[B131] Gayet M Meunier FJ Premiére découverte de Gymnotiformes fossiles (Pisces, Ostariophysi) dans le Miocène supérieur de Bolivie CR Acad Sci Paris 1991 313 4 471 476

[B132] Gayet M Meunier FJ Arratia G, Kapoor BG, Chardon M, Diogo R Paleontology and palaeobiogeography of catfishes Catfishes 2003 Enfield, NH: Science Publishers 491 522

[B133] Wilson MVH Brinkman DB Neuman AG Cretaceous Esocoidei (Teleostei): early radiation of the pikes in North American fresh waters J Paleontol 1992 66 839 846

[B134] Tyler JC Sorbini L New superfamily and three new families of tetraodontiform fishes from the Upper Cretaceous: the earliest and most morphologically primitive plectognaths Smith Contr Paleobio 1996 82 1 59

[B135] Benton MJ Donoghue PCJ Asher RJ Hedges SB, Kumar S Calibrating and constraining molecular clocks Timetree of life 2009 Oxford, UK: Oxford University Press 35 86

