# Supplementary figures and images for: Evolutionary history of Otophysi (Teleostei), a major clade of the modern freshwater fishes: Pangaean origin and Mesozoic radiation
Source: BMC Evol Biol. 2011 Jun 22;11:177. doi: 10.1186/1471-2148-11-177 (PMC3141434; doi:10.1186/1471-2148-11-177)

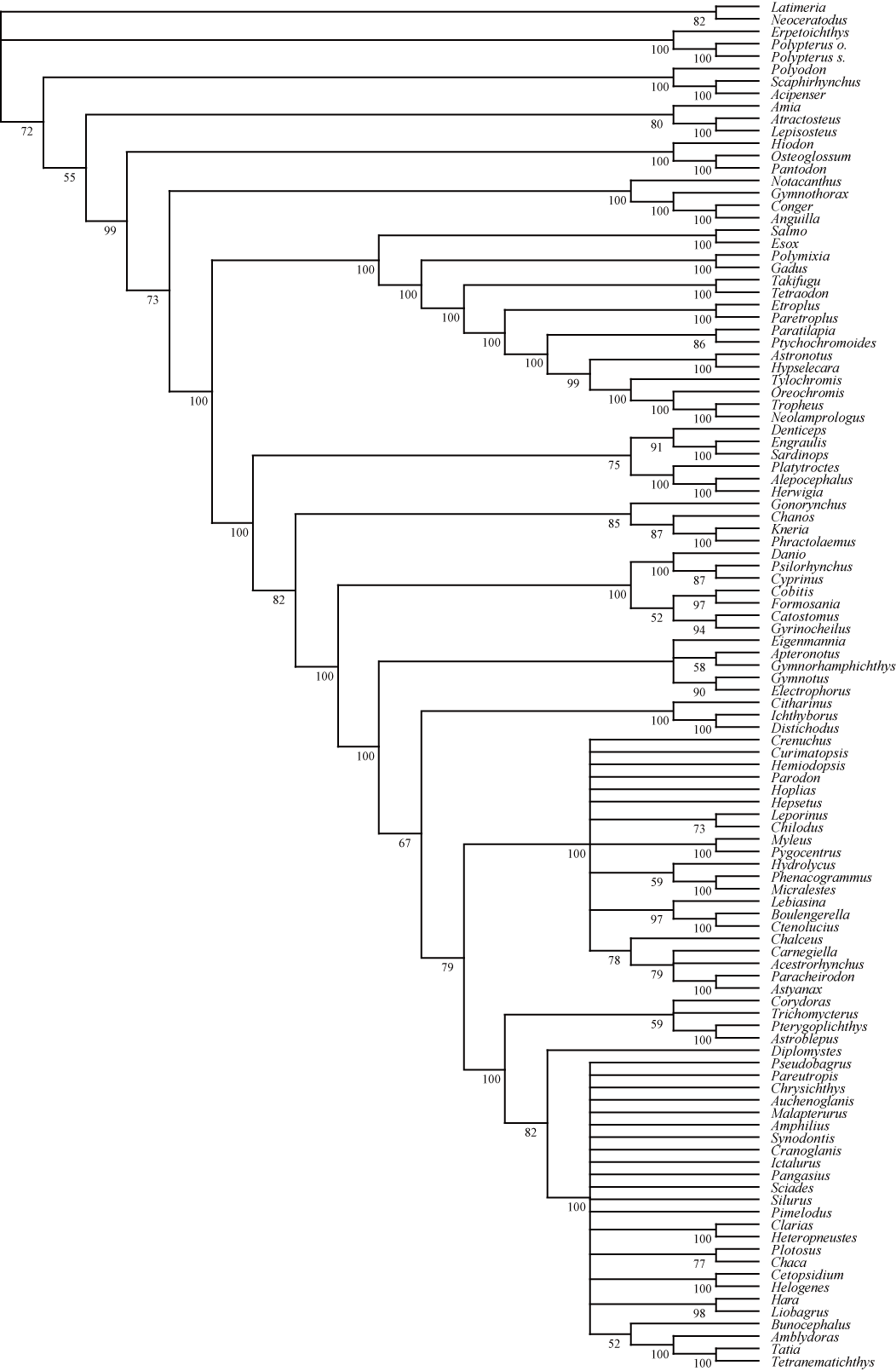


Additional file 1. A cladogram showing all nodes with < 50% BSPs in Figure 3 collapsed to polytomy.

Supplement: Additional file 1 — A cladogram showing all nodes with < 50% BSPs in Figure 3 collapsed to polytomy. [file 1471-2148-11-177-S1.DOC]
