# Supplementary material for: Evolutionary history of Otophysi (Teleostei), a major clade of the modern freshwater fishes: Pangaean origin and Mesozoic radiation
Source: BMC Evol Biol. 2011 Jun 22;11:177. doi: 10.1186/1471-2148-11-177 (PMC3141434; doi:10.1186/1471-2148-11-177)
Supplement: Additional file 2 — Summary of MCMC samples for node ages (means and 95% credible intervals) in the independent-rates (IR) and autocorrelated-rates (AR) analyses using MCMCTREE-HS [63]. Results from the two data sets (12nRTn and 123a) are shown separately. For node numbers, see tree at the end of this file. [file 1471-2148-11-177-S2.DOC]

**Additional file 2-1.**  Summary of MCMC samples based on the nucleotide data set (12_n_RT_n_) for node ages (posterior means and 95% credible intervals) in the independent-rates (IR) analyses using MCMCTREE-HS. For node numbers, see a tree at the end of this file.

—————————————————————————————————

Node Mean 95% 95% Node Mean 95% 95%

(lower) (upper) (lower) (upper)

—————————————————————————————————

111 444.5 419.1 471.7 151 178.5 149.6 208.0

112 425.2 401.4 449.4 152 247.7 227.1 268.4

113 380.2 359.7 394.6 153 159.5 129.7 186.3

114 134.5 130.0 143.5 154 134.6 106.0 161.7

115 28.4 15.6 42.8 155 109.5 83.4 135.9

116 361.5 338.9 381.3 156 149.8 120.5 177.9

117 341.0 313.5 365.6 157 128.5 97.8 157.9

118 50.3 29.9 73.3 158 125.7 95.4 156.5

119 334.0 311.9 357.0 159 225.5 206.0 245.4

120 274.2 236.4 309.3 160 188.7 166.0 211.5

121 194.9 152.3 236.9 161 165.0 137.5 190.0

122 325.9 302.2 347.7 162 182.2 159.3 205.4

123 256.7 223.3 291.8 163 169.7 145.4 193.6

124 188.6 156.2 224.7 164 220.2 201.2 240.2

125 154.7 120.2 189.4 165 159.8 123.8 190.4

126 293.2 271.2 315.7 166 81.0 52.6 112.3

127 266.2 243.3 290.2 167 216.3 198.0 236.6

128 203.8 154.1 243.1 168 192.4 171.9 212.8

129 214.9 189.6 237.9 169 188.8 168.2 209.2

130 168.2 138.3 198.5 170 170.7 149.3 192.1

131 179.4 159.0 201.9 171 119.0 95.9 140.3

132 87.7 61.1 113.5 172 179.9 159.3 200.6

133 125.5 116.6 135.5 173 171.6 150.4 192.6

134 91.5 86.0 95.4 174 163.7 142.3 185.4

135 117.3 112.0 125.2 175 100.0 76.5 122.2

136 107.3 95.7 119.2 176 187.8 167.9 209.3

137 106.3 100.0 114.3 177 183.3 163.0 204.6

138 74.1 57.7 91.4 178 178.5 157.0 199.6

139 71.9 56.1 87.8 179 160.4 132.2 184.2

140 50.3 36.7 64.5 180 162.2 137.8 184.1

141 26.8 17.3 37.1 181 98.7 73.8 122.1

142 264.8 243.4 286.2 182 173.7 150.7 196.0

143 258.5 236.2 280.4 183 35.8 19.9 53.5

144 232.2 205.4 259.3 184 172.0 149.6 195.2

145 132.7 98.2 167.6 185 157.9 130.3 183.2

146 55.9 38.3 74.7 186 158.4 132.7 182.5

147 39.0 24.4 54.7 187 138.9 112.5 166.8

148 261.4 239.6 282.1 188 179.9 161.8 198.1

149 254.4 231.7 276.4 189 170.3 149.7 189.7

150 230.6 204.6 256.4 190 147.0 121.8 172.5

—————————————————————————————————

—————————————————————————————————

Node Mean 95% 95% Node Mean 95% 95%

(lower) (upper) (lower) (upper)

—————————————————————————————————

191 161.1 137.9 182.7 206 102.6 85.2 119.7

192 169.6 152.6 187.5 207 87.1 67.9 104.9

193 142.8 127.8 158.9 208 128.6 113.3 144.1

194 138.6 124.0 155.8 209 126.1 110.9 142.3

195 132.5 116.5 150.4 210 120.3 103.9 136.6

196 109.4 90.6 130.6 211 104.7 87.3 122.8

197 135.5 120.0 152.6 212 120.3 105.6 137.2

198 105.0 87.0 123.7 213 110.3 94.0 126.5

199 115.5 98.0 133.9 214 102.0 85.5 118.9

200 139.1 124.5 155.5 215 111.9 95.2 128.4

201 124.7 106.4 144.6 216 99.0 81.7 117.6

202 135.2 120.5 150.9 217 123.7 91.6 158.2

203 132.5 117.7 148.8 218 74.2 47.7 104.0

204 63.6 36.1 95.8 219 418.0 365.5 465.8

205 120.0 103.3 136.3 —————————————————————————————————

**Additional file 2-2.**  Summary of MCMC samples based on the amino acid data set (123_a_) for node ages (posterior means and 95% credible intervals) in the independent-rates (IR) analyses using MCMCTREE-HS. For node numbers, see a tree at the end of this file.

—————————————————————————————————

Node Mean 95% 95% Node Mean 95% 95%

(lower) (upper) (lower) (upper)

—————————————————————————————————

111 442.3 417.6 470.8 151 127.0 76.3 183.8

112 422.3 395.6 448.6 152 230.9 196.8 264.8

113 373.3 347.7 392.2 153 144.9 110.4 185.8

114 145.4 130.5 187.4 154 116.2 78.1 156.6

115 70.9 16.7 128.3 155 92.6 58.3 135.6

116 351.2 318.9 378.8 156 131.2 95.2 169.2

117 324.6 288.1 363.1 157 106.9 68.6 148.0

118 76.1 24.1 139.7 158 105.6 67.1 147.4

119 314.7 276.5 349.5 159 208.6 177.4 241.3

120 231.0 163.7 292.5 160 159.5 124.6 199.2

121 154.7 114.7 224.0 161 126.4 85.6 165.9

122 305.4 267.0 341.7 162 140.1 103.6 180.5

123 220.5 144.7 288.4 163 119.1 79.7 158.6

124 134.7 86.6 199.7 164 200.1 169.9 231.8

125 102.5 60.6 163.7 165 139.7 96.9 183.7

126 276.4 239.7 312.5 166 77.7 35.9 122.7

127 242.7 203.8 282.7 167 195.8 165.7 227.0

128 146.8 82.6 224.2 168 175.3 143.8 212.7

129 203.6 168.0 245.7 169 167.3 136.1 204.8

130 144.9 100.8 199.4 170 140.4 107.2 180.8

131 171.4 143.6 209.7 171 96.3 63.9 134.5

132 66.1 39.9 108.3 172 156.0 125.2 193.4

133 135.9 120.5 156.7 173 141.9 112.4 180.3

134 90.3 85.3 95.0 174 128.8 97.3 166.2

135 122.7 112.7 137.2 175 84.0 51.6 120.2

136 101.0 69.7 125.0 176 165.2 133.0 203.0

137 110.6 101.1 119.8 177 155.9 126.4 189.9

138 78.8 51.3 99.8 178 146.0 116.2 180.2

139 86.7 62.4 105.0 179 116.9 76.6 155.6

140 69.1 36.0 92.8 180 125.0 93.9 158.6

141 44.7 17.3 76.1 181 81.5 49.5 116.7

142 254.8 217.7 290.6 182 139.3 111.3 171.5

143 219.1 156.3 268.7 183 60.2 16.0 114.5

144 169.4 114.8 235.1 184 133.6 105.2 165.4

145 91.4 59.6 144.4 185 116.1 78.2 152.6

146 97.1 55.9 144.6 186 114.3 82.4 148.5

147 77.0 35.3 124.0 187 100.3 67.3 136.8

148 246.0 209.9 281.6 188 173.1 145.6 202.1

149 229.7 174.2 271.8 189 153.5 118.2 187.7

150 180.1 119.0 236.3 190 118.1 75.3 158.0

—————————————————————————————————

—————————————————————————————————

Node Mean 95% 95% Node Mean 95% 95%

(lower) (upper) (lower) (upper)

—————————————————————————————————

191 125.9 82.9 165.4 206 90.7 64.7 119.0

192 163.0 136.7 191.3 207 70.5 42.0 97.0

193 145.0 121.6 172.4 208 126.0 101.9 154.0

194 138.1 113.9 166.1 209 119.7 92.9 148.5

195 121.8 90.5 153.0 210 106.2 77.8 137.5

196 94.7 63.0 130.8 211 86.6 57.1 120.5

197 127.7 101.4 156.8 212 116.9 90.1 145.5

198 94.0 63.3 129.8 213 104.8 77.6 134.4

199 97.6 66.4 131.1 214 86.5 59.4 117.9

200 141.0 117.2 168.0 215 101.6 72.2 132.4

201 128.8 93.1 159.5 216 89.6 61.4 121.3

202 132.8 109.4 159.5 217 141.3 90.8 213.6

203 125.7 101.6 152.4 218 89.2 46.9 138.8

204 70.7 25.9 109.7 219 340.2 246.0 453.0

205 109.8 84.2 137.6 —————————————————————————————————

**Additional file 2-3.**  Summary of MCMC samples based on the nucleotide data set (12_n_RT_n_) for node ages (posterior means and 95% credible intervals) in the autocorrelated-rates (AR) analysis using MCMCTREE-HS. For node numbers, see a tree at the end of this file.

—————————————————————————————————

Node Mean 95% 95% Node Mean 95% 95%

(lower) (upper) (lower) (upper)

—————————————————————————————————

111 439.5 417.7 466.9 151 176.7 150.9 202.8

112 422.2 400.2 443.9 152 240.9 222.3 257.7

113 384.4 366.0 393.8 153 191.8 172.1 209.7

114 144.7 130.3 176.5 154 162.8 140.4 185.3

115 84.9 53.9 109.6 155 142.9 115.7 167.4

116 364.4 344.7 378.8 156 179.8 160.8 197.0

117 344.2 321.5 363.3 157 166.6 146.0 185.0

118 85.8 38.1 139.3 158 167.3 147.1 185.7

119 332.9 313.0 349.5 159 216.6 199.5 231.9

120 291.5 267.7 312.4 160 180.7 163.6 196.7

121 190.5 129.2 238.5 161 161.5 142.9 178.4

122 322.7 302.5 340.0 162 176.8 160.1 192.8

123 267.3 242.7 292.2 163 166.6 149.4 183.8

124 204.2 176.2 230.3 164 212.2 195.1 227.3

125 176.9 148.0 204.1 165 169.2 149.9 187.6

126 288.7 269.2 307.0 166 94.9 72.7 116.2

127 262.7 243.9 281.2 167 208.4 191.4 223.4

128 229.7 199.8 252.4 168 196.6 180.1 211.0

129 215.4 198.5 233.6 169 192.4 175.4 207.1

130 170.9 143.0 195.2 170 174.0 157.0 189.9

131 173.7 159.6 189.0 171 105.2 84.7 124.7

132 87.8 63.2 117.7 172 184.0 167.6 198.7

133 123.7 115.8 133.5 173 175.0 157.9 190.6

134 89.6 85.2 94.9 174 168.2 151.8 184.0

135 116.0 112.2 123.7 175 91.2 71.3 111.3

136 110.9 104.0 119.7 176 192.8 176.1 207.5

137 106.5 100.9 114.8 177 189.7 173.5 204.2

138 61.5 45.8 77.6 178 183.9 167.7 198.9

139 65.7 54.2 78.2 179 169.5 152.7 185.3

140 54.1 44.7 64.9 180 167.8 151.2 183.4

141 19.5 12.5 28.5 181 92.5 69.6 113.1

142 258.2 239.3 276.0 182 182.9 166.8 197.3

143 253.5 233.7 272.7 183 43.7 20.0 93.9

144 230.9 208.3 252.1 184 181.6 165.7 196.2

145 127.2 76.8 169.6 185 169.3 152.4 185.1

146 96.7 77.7 117.5 186 169.8 153.4 185.5

147 71.5 52.7 92.8 187 155.4 137.2 172.2

148 256.1 237.2 273.9 188 169.8 154.7 185.7

149 250.4 231.0 268.6 189 162.1 146.3 178.5

150 234.0 214.7 253.0 190 148.1 129.7 166.6

—————————————————————————————————

—————————————————————————————————

Node Mean 95% 95% Node Mean 95% 95%

(lower) (upper) (lower) (upper)

—————————————————————————————————

191 156.8 140.3 173.5 206 85.5 74.0 96.7

192 159.1 144.4 174.3 207 69.4 56.6 82.3

193 120.2 108.9 132.3 208 107.7 96.1 119.0

194 116.1 104.8 129.0 209 105.0 93.4 116.3

195 111.9 100.5 125.1 210 101.5 89.7 112.8

196 92.9 80.1 107.0 211 86.3 72.8 98.3

197 113.8 102.0 127.0 212 100.6 88.9 112.1

198 86.0 72.2 98.4 213 94.3 83.0 105.7

199 95.2 82.7 109.8 214 85.9 72.9 97.7

200 116.5 105.2 128.8 215 93.8 82.3 105.3

201 106.3 93.7 119.5 216 83.7 70.2 95.5

202 112.1 101.0 123.7 217 107.7 78.5 171.1

203 110.2 98.9 122.0 218 49.6 31.6 72.5

204 48.7 26.8 75.2 219 416.6 370.2 458.8

205 98.2 86.0 111.1 —————————————————————————————————


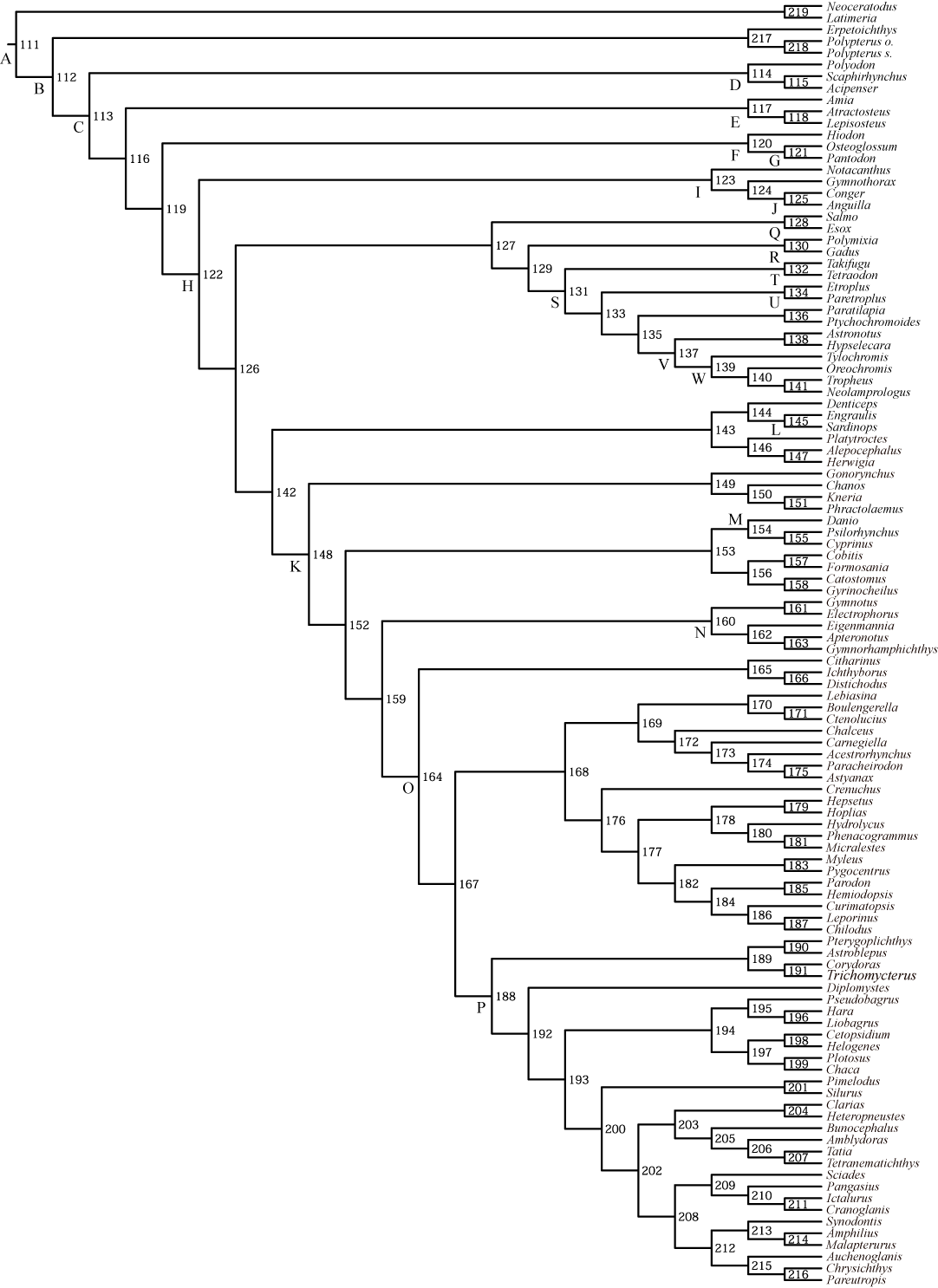


Additional file 2-4. Node numbers in the timetree.
