# Supplementary material for: Evolutionary history of Otophysi (Teleostei), a major clade of the modern freshwater fishes: Pangaean origin and Mesozoic radiation
Source: BMC Evol Biol. 2011 Jun 22;11:177. doi: 10.1186/1471-2148-11-177 (PMC3141434; doi:10.1186/1471-2148-11-177)
Supplement: Additional file 3 — Timetree derived from the Bayesian relaxed-molecular clock method (non-ostariophysan portions also shown). Upper (maximum) and lower (minimum) time constraints used in this study are shown by arrowheads with corresponding nodes connected by dotted lines. All marine species are indicated by asterisks. [file 1471-2148-11-177-S3.DOC]

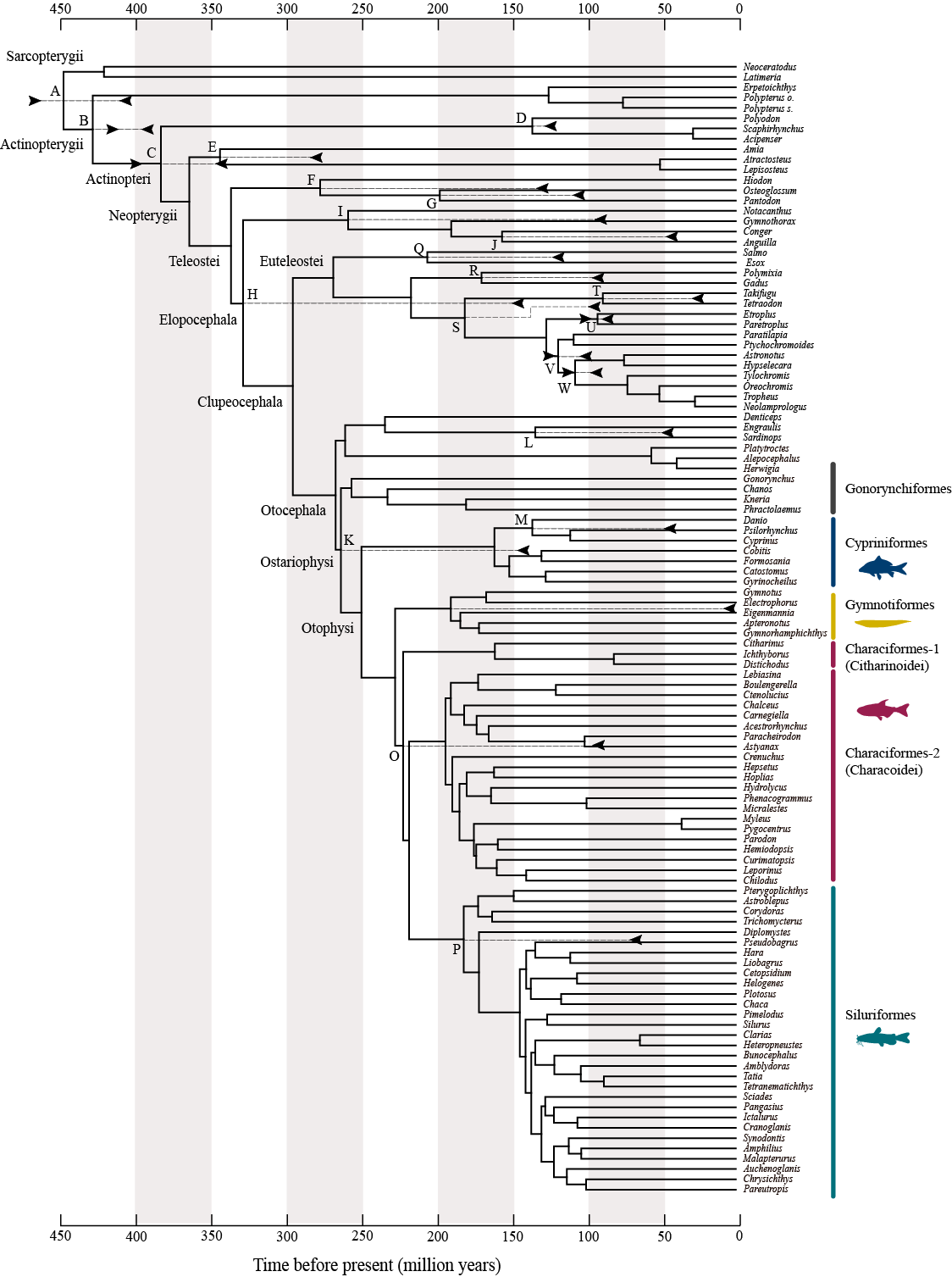


Additional file 3. Timetree derived from the Bayesian relaxed-molecular clock method. All time constraints are shown with arrowheads with dotted lines indicating corresponding nodes.
